# Age-Based Developmental Biomarkers in Eye Movements: A Retrospective Analysis Using Machine Learning

**DOI:** 10.3390/brainsci14070686

**Published:** 2024-07-09

**Authors:** Melissa Hunfalvay, Takumi Bolte, Abhishek Singh, Ethan Greenstein, Nicholas P. Murray, Frederick Robert Carrick

**Affiliations:** 1 RightEye LLC., 6107A, Suite 400, Rockledge Drive, Bethesda, MD 20814, USA; takumi@righteye.com (T.B.); abhishek27297@gmail.com (A.S.); 2 Biological Engineering, Massachusetts Institute of Technology, Cambridge, MA 02139, USA; 3 Washington University in St. Louis, 1 Brookings Dr., St. Louis, MO 63130, USA; ethangreenstein@wustl.edu; 4 Visual Motor Laboratory, Department of Kinesiology, East Carolina University, Greenville, NC 27858, USA; murrayni@ecu.edu; 5 Neurology, University of Central Florida College of Medicine, Orlando, FL 23816, USA; drfrcarrick@post.harvard.edu; 6 Centre for Mental Health Research in Association with University of Cambridge, Cambridge CB3 9AJ, UK; 7 MGH Institute of Health Professions, Boston, MA 02129, USA; 8 Carrick Institute Neurology, Cape Canaveral, FL 32920, USA

**Keywords:** eye movements, machine learning, eye tracking, lifespan development

## Abstract

This study aimed to identify when and how eye movements change across the human lifespan to benchmark developmental biomarkers. The sample size comprised 45,696 participants, ranging in age from 6 to 80 years old (*M* = 30.39; *SD* = 17.46). Participants completed six eye movement tests: Circular Smooth Pursuit, Horizontal Smooth Pursuit, Vertical Smooth Pursuit, Horizontal Saccades, Vertical Saccades, and Fixation Stability. These tests examined all four major eye movements (fixations, saccades, pursuits, and vergence) using 89 eye-tracking algorithms. A semi-supervised, self-training, machine learning classifier was used to group the data into age ranges. This classifier resulted in 12 age groups: 6–7, 8–11, 12–14, 15–25, 26–31, 32–38, 39–45, 46–53, 54–60, 61–68, 69–76, and 77–80 years. To provide a descriptive indication of the strength of the self-training classifier, a series of multiple analyses of variance (MANOVA) were conducted on the multivariate effect of the age groups by test set. Each MANOVA revealed a significant multivariate effect on age groups (*p* < 0.001). Developmental changes in eye movements across age categories were identified. Specifically, similarities were observed between very young and elderly individuals. Middle-aged individuals (30s) generally showed the best eye movement metrics. Clinicians and researchers may use the findings from this study to inform decision-making on patients’ health and wellness and guide effective research methodologies.

## 1. Introduction

Our eyes are often said to be windows to our souls. Eye movements are highly sensitive representations of brain function and, therefore, of an individual’s health and wellness [1]. Eye movements are measured noninvasively using eye-tracking technology that provides objective ways to understand neural pathways. 

Some eye movements have clinical significance, such as tremors related to early-stage Parkinson’s disease [2]. Other eye movements provide information regarding the muscle coordination of the eyes that allows individuals to see the world in three dimensions [3]. Other eye movements may reflect expertise in a skill, such as that of a sport or diagnostic procedure (e.g., an experienced clinician examining a radiological chest X-ray) [4].

Although eye-tracking hardware is becoming ubiquitous, and eye movement research is plentiful, limitations in setting benchmarks exist. Perhaps the most critical limitation is understanding how eye movements change across the lifespan. To date, researchers and clinicians struggle with answering fundamental questions, such as: What should be considered “functional” eye movement results across the lifespan? When do movements of the eye significantly change? How do they change? Which eye movements change? Answers to these questions will provide critical biomarkers to aid in understanding a person’s health and wellness. Furthermore, such markers can assist in rapid triage, informing treatment, and aiding in monitoring recovery.

As of the writing of this paper, twenty-one studies were found that examined eye movements as we age. In examining these studies, three main limitations were identified. 

The first limitation is the small sample sizes. Normative data studies should be obtained from very large samples, typically many thousands of diverse participants [5]. Of the twenty-one studies found, sample sizes range from groups in the single digits (e.g., Scherf et al., *n* = 9 for 10–13-year-olds, *n* = 13 for 14–17-year-olds) [6] to the largest sample size of 2993 participants in a group of 5–65-year-olds (e.g., Murray et al., 2019) [7].

A second limitation includes the type of eye movements measured in these studies. The four major eye movements are fixations, saccades, pursuits, and vergence. Fixations are stopping points that hold an image on the fovea for detailed examination [1]. Saccades are quick ballistic eye movements that reorient the eye to places across the field of view. Smooth pursuits eye movements follow an object through space, such as the motion of tracking a ball. Vergence eye movements are where the eyes move in oppositive directions so that images of a single object are placed or held simultaneously as the object moves closer or further away [1].

Most studies focus solely on saccadic eye movements. There are a few exceptions, such as Kullman et al., who measured saccades and pursuits [8]; Mokler and Fischer, who measured saccades and fixations [9]; and Gould et al., who measured fixation alone [10]. The limitation in not measuring all of the major eye movements leads to an incomplete picture of the visual and cognitive aspects that eye movements pose to health and wellness. 

The final, third major limitation of normative data studies to date is, indeed, the lack of widespread examination of eye movements across the entire lifespan. Studies have shown differences in specific eye movements at certain ages. For example, Fukushima et al. observed that saccadic reaction time plateaued by 12 years of age [11]. Others, like Kullman et al., have shown no age differences when comparing 18–21-year-olds (late adolescents) and 21–45-year-olds [12]. Lenzenweger and O’Driscoll stated that they “limited the age to one group of 18–45 years to avoid potential age-related artifacts” [13].

Murray et al. attempted to identify distinct groups by age using an unsupervised machine learning method [7]. With a sample size of 2993, the model-based clustering used expectation maximization (EM) algorithm analysis. The results identified five distinct age group clusters: 5–8, 9–16, 17–28, 29–52, and 53–62.

Although an important step in data-driven age group determination of eye movements, Murray et al. still present two main limitations [7]. First, there was a relatively small sample size [7]. Second, there was a limited age range, especially regarding a lack of elderly adults [7]. Therefore, per Campbell’s assertion that “normative data studies are typically obtained from very large, randomly selected representative samples of the whole population” [5], the purpose of this study is multifaceted. First, this study aims to allow for eye movement data to drive the identification of age groups using a semi-supervised machine learning methodology. The second purpose is to include a large volume of age-based eye movement data from tens of thousands of participants (*n* = 46,655) across the entire human lifespan to increase the generalizability of the results. The third purpose is to include the four major eye movements (fixations, saccades, pursuits, and vergence). Therefore, this study aims to identify age-based developmental biomarkers for eye movements that can be used as milestones for clinicians and researchers to inform decision-making on patients’ health and wellness and to guide future research methodologies. 

## 2. Materials and Methods

### 2.1. Participants

Participants were recruited from 804 sites containing RightEye Vision Systems. The sites were optometry practices throughout the United States where participants had come for a clinical visit or annual exam. Selection criteria included participants who had completed all six eye movement tests, which include Circular Smooth Pursuit (CSP), Horizontal Smooth Pursuit (HSP), Vertical Smooth Pursuit (VSP), Horizontal Saccades (HS), Vertical Saccades (VS), and Fixation Stability (FS). 

Participants were excluded from this study if they had eyelash impediments, had consumed drugs or alcohol within 24 h of testing, tested positive for strabismus, or failed to pass all 9 points of calibration. 

This sample included 45,696 participants ranging in age from 6 to 80 years old. The mean age was 30.39 (*SD* = 17.46) years. 

Gender was reported in 51.45% (*n* = 23,557) of the participants. Of the data that included gender, 11,871 participants were male (50.39%) and 11,686 participants were female (49.61%). Of the total 45,696 participants, 42.38% (*n* = 19,366) reported their race and ethnicity, with the majority identifying as White (*n* = 13,465, 69.53%). The next largest ethnic group was Asian at 5.48% (*n* = 1063), then Latin American (*n* = 982, 5.07%), then Black (*n* = 796, 4.11%). 

Handedness was reported by 51.45% (*n* = 23,109) of the participants, most of whom were right-handed (*n* = 20,467, 88.57%). Of the total, 2010 (8.70%) were left-handed and 632 (2.73%) reported being ambidextrous. 

This study was conducted in accordance with the tenets of the Declaration of Helsinki. The study protocols were approved by the Institutional Review Board of East Carolina University (IRB UMCIRB 13-002660). All data used for the analysis are anonymized. 

### 2.2. Testers

Board-certified (the American Board of Optometry) optometrists conducted the testing. The clinician was trained in using the RightEye vision system and became a certified RightEye provider.

### 2.3. Apparatus

Stimuli were presented using the RightEye tests on a Tobii I15 vision 15 monitor fitted with a Tobii 90 Hz remote eye tracker and a Logitech (model Y-R0017) wireless keyboard and mouse. The accuracy of the Tobii eye tracker was 0.4° within the desired headbox of 32 cm × 21 cm at 55 to 60 cm from the screen. 

### 2.4. Testing Procedure

Testers were required to complete and pass the RightEye Basic Training Course designed to teach them the appropriate test setup and data collection procedures. This setup included seating participants in a stationary (nonwheeled) chair that could not be adjusted in height. Participants sat in front of a desk in a quiet, private room. Participants’ heads were unconstrained. 

**Figure 1:**
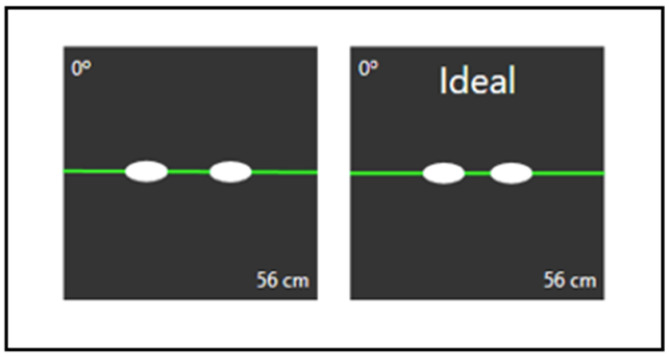
Head box guidance system showing real-time head box adjustments and ideal participant location to RightEye vision system. Measurement in centimeters (cm).

For standardization of testing, participants were asked to sit in front of the eye-tracking system at an exact measured distance of 56 cm (ideal positioning within the headbox range of the eye tracker). This was validated in real time using the RightEye head box guidance system (Figure 1).

Participants then calibrated using a standard 9-point calibration task. If all 9 calibration points were passed, participants were tested on the oculomotor tasks. 

### 2.5. Oculomotor Tasks

Calibration Task: This consisted of nine points of gaze, each the size of one degree, and presented one at a time for 2 s. The visual targets appeared in random locations across the screen. The calibration task took 18 s to complete. At the task’s end, the participant would have effectively viewed the 9 sections of the screen. The purpose of the calibration task was to enable the eye tracker to turn information about the location of the centers of the pupil and corneal reflection (pixel coordinates) into gaze locations on the screen. This information elicited pupil measurements (e.g., pupil size) and was used to track gaze throughout subsequent tests.
Pursuit Tasks: This included Circular Smooth Pursuit (CSP), Horizontal Smooth Pursuit (HSP), and Vertical Smooth Pursuit (VSP). Participants were verbally asked to “follow the dot on the screen as accurately as possible with your eyes”. The dot was 0.2 degrees in diameter and moved at a speed of 25.13 degrees of visual angle per second. The tests were taken with a black background with white dots and lasted 20 s. For the CSP test, the diameter of the circle’s movement was 20 degrees. For the HSP test, the dot moved 15 degrees left and 15 degrees right of the central point (totaling 30 degrees horizontally). For the VSP test, the dot moved 11 degrees up and 11 degrees down from the central point (totaling 22 degrees).
Saccade Tasks: This included the horizontal self-paced saccades test (HS) and vertical self-paced saccades test (VS). Participants were asked to look at a countdown from 3 to 1 in the center of the screen before moving their eyes back and forth between 2 dots. Their goal was to “target each dot” on the left and right for the HS test (up and down for the VS test) on the screen as quickly and accurately as possible. The test lasted 10 s.
Fixation Task: This task included the Fixation Stability (FS) test. Participants were required to view three targets, presented one at a time, for seven seconds each, with a break of three seconds between targets. Before each target was presented, identical verbal instructions were given to every participant: “Move your eyes to the center of the target. Keep your eyes as still as possible until the target disappears”. The tester then asked, “Are you looking at the center of the target”? Once the participant confirmed with a verbal “Yes”, the tester pressed the spacebar, and the 7 second time began. Targets included a 1° cross, a 1° filled circle, and a small 4-point diamond (3° point-to-point separation) using dimensions as in the Humphrey Field Analyzer (Carl Zeiss Meditec, Dublin, CA, USA) and specified in Bellmann et al. [14].

### 2.6. Eye-Tracking Algorithms 

Eighty-nine eye-tracking algorithms were derived from the seven different oculomotor tasks. CSP contributed 17 variables, HSP contributed 18 variables, VSP contributed 13 variables, HS contributed 14 variables, VS contributed 12 variables, FS contributed 13 variables, and calibration contributed 2 pupil variables. Table A1, Table A2, Table A3, Table A4, Table A5, Table A6 and Table A7 in Appendix A show the variables calculated, including the eye movement classification category being measured (vergence, saccade, fixation, pursuit, blink, or pupil), the eye being measured (left, right, or both eyes), and the name and definition of the algorithm for each of the oculomotor tasks.

**Figure 2:**
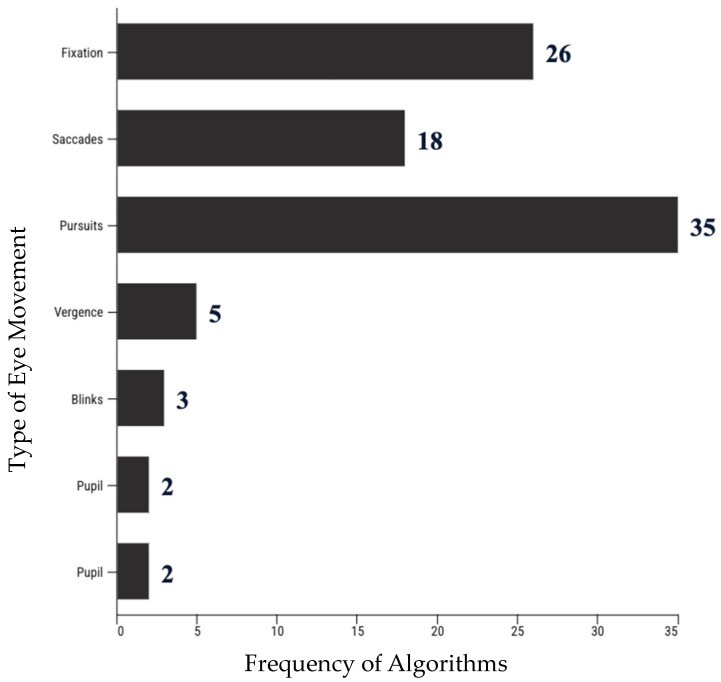
Frequency of eye movement, blink, and pupil algorithms.

In summary, the tasks included all major eye movements (fixations, saccades, pursuits, and vergence) and two additional eye measures in pupil and blink algorithms. Figure 2 shows the type of eye movement and the number of associated algorithms.

### 2.7. Data Analysis

Analysis was conducted in Jupyter (Version 7.0.6) using Python (Version 3.5) and Scikit Learn (Version 0.21). A semi-supervised, self-training, machine learning classifier was used to group data into age ranges. Understanding the data groups is enhanced by using these algorithms because of their inherent ability to analyze complex, large datasets and derive meaningful patterns in the data [15].

Random forest classifier (RFC) was used as the base classifier. RFCs are commonly used ensemble machine learning (ML) algorithms that use multiple decision trees to reach a single result [16]. Each iteration of the decision tree is developed using a random subset of the eye-tracking variables [17]. 

The base classifier in a self-training classifiers (STCs) provides uncertainty estimates for predictions. The STC algorithm was proposed by Yarowsky (1995) [18]. The STC allows for supervised classifiers to act semi-supervised to learn from unlabeled data. It is an iterative algorithm that predicts pseudo-labels for the unlabeled data and adds them to the training set. The algorithm continues iterating until a stop condition is reached. The stop conditions included a maximum depth of 10, using maximum features of the square root, with maximum iterations of 200 and a threshold of 95%. 

Prior to running the RFC, twenty-three ages (measured in years) were removed (unlabeled), and the non-labeled dataset was formed. The STC acts as a wrapper on the base classifier, enabling it to learn from partially labeled data, making the machine learning algorithm semi-supervised [15]. To determine the transferability of the data, 80% was used for training and 20% for testing. Accuracy, defined as the model’s ability to correctly classify the age of each participant in the unlabeled test data, was used to evaluate it. 

Upon completion of the STC, a series of MANOVAs were conducted on the multivariate effect of the age groups by test set (e.g., CSP, HSP, VSP, HS, VS, and FS variables). MANOVAs were conducted to provide a descriptive indication of the strength of the STC. The probability was set to *p* < 0.05. 

## 3. Results

**Figure 3:**
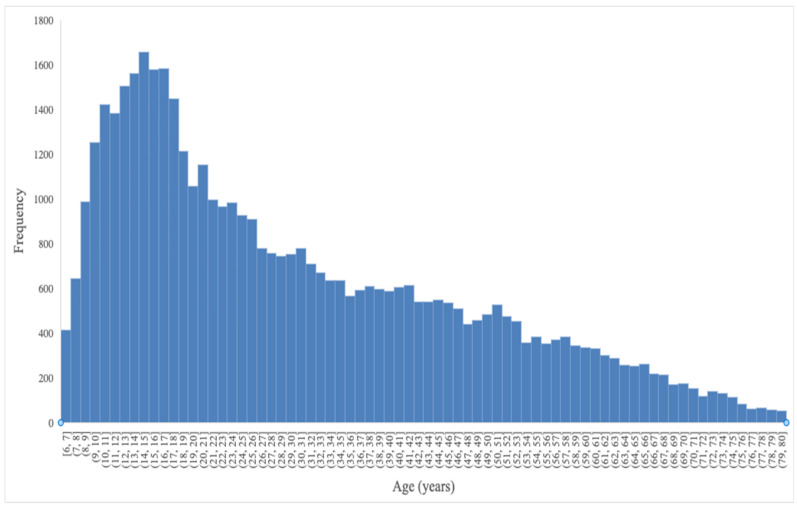
Age distribution histogram.

The sample size was 45,696, and participants ranged from 6 to 80 years old (Figure 3). The mean age (SD) was 30.39 (17.46) years. As shown in Figure 3, the distribution is skewed right positively; therefore, data are also reported as median and interquartile ranges (IQRs) [19]. The median (IQR) was 26.00 (16.00–43.00) years.

The STC resulted in 12 age groups: 6–7, 8–11,12–14, 15–25, 26–31, 32–38, 39–45, 46–53, 54–60, 61–68, 69–76, and 77–80 years. Table 1 shows the descriptive, age, and gender statistics of each age group defined by the STC. The interquartile range (IQR) between the 25th and 75th percentiles and the middlemost number (median) reported as age data are positively skewed [19].

**Figure 4:**
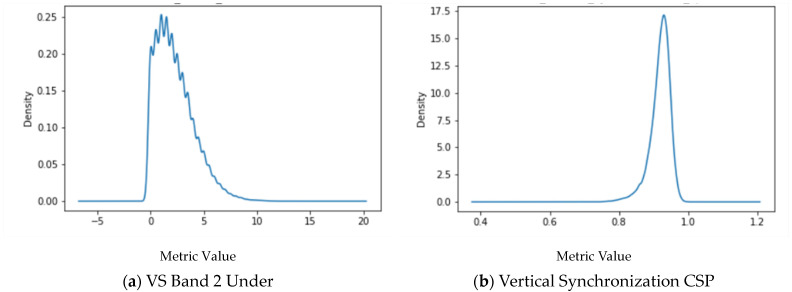
The left graph (**a**) shows positive skewness in the Vertical Saccades test variable of Band 2 Under. The right graph (**b**) shows negative skewness in the Vertical Synchronization Circular Smooth Pursuit test variable.

Skewness: The skewness range was between −10 and +10 for all ET variables. Figure 4 shows two representative samples of the skewness of eye movement variables.

The positive skew of VS Band 2 Under is depicted in Figure 4a. As such, more participants had lower values for this variable. In other words, more participants were performing below the average. As the longer right tail indicates, fewer participants performed above the average. Figure 4b reveals an opposite trend. Depicted in 4b, the Vertical Synchronization CSP, a negative skew was seen. In other words, more participants were performing above the average. As the longer left tail indicated, fewer participants performed below average. Due to the skewness of the data, the median and IQR for each eye tracking variable by age group within each oculomotor task were used to represent the measures of central tendency and variability (see Appendix B, Table A8, Table A9, Table A10, Table A11, Table A12, Table A13 and Table A14).

The STC correctly classified the age group of participants 94.67% of the time. To provide a descriptive indication of the strength of the STC, a series of MANOVAs were conducted on the multivariate effect of the age groups by test set (e.g., CSP, HSP, VSP, HS, VS, and FS variables). Each MANOVA revealed a significant multivariate effect on age groups (See Table 2), thus indicating reasonable support for the STC model. This significant multivariate effect on age groups (*p* < 0.001) upholds the robustness of our model.

## 4. Discussion

This study aimed to identify when and how eye movements change across the human lifespan to benchmark developmental biomarkers. The semi-supervised machine learning model stratified individuals into 12 age groups based on eye movement data with high accuracy (94.67%). Follow-up MANOVAs were used to indicate the strength of each age group; the results indicated a high level of significance (*p* < 0.001). 

These findings reveal age-based developmental biomarkers associated with eye movements across many participants (*n* = 46,655) and a vast age range (6 to 80), adding to the current body of knowledge by enhancing the generalizability of age-based, eye-tracking biomarkers [7,8,11,12].

An interesting observation regarding the age groups identified through the eye movement variables is that young and elderly individuals have smaller age ranges when compared to other stages of life. For example, Group 1 includes 6-to-7-year-olds, a two-year age span. Group 2 includes 8-, 9-, 10- and 11-year-olds, a 4-year age span. Group 12 includes 77-, 78-, 79-, and 80-year-olds, again a 4-year age span. In the early stages of development and later stages of decline, the differences in eye movements related to age change more rapidly [1]. In early adulthood, from 15 to 25, there is little change, according to our results. Then, in middle age, change seems to occur about every five or six years, as seen by the age groups 26 to 31 (Group 5), 32 to 38 (Group 6), and 39 to 45 (Group 7). These results are consistent with other life span development research, such as that which has found a linear reduction in processing speed as we age [20] and that our thinking abilities appear to peak around age 30 on average and then decline subtly with age [21].

This study shows that age affects eye movement behaviors in broad and significant ways, consistent with the current body of literature. The four major eye movements (fixations, saccades, pursuits, and vergence) were examined via the different tests (CSP, HSP, VSP, HS, VS, and FS variables). Follow-up MANOVAs revealed highly significant differences across all testing protocols.

**Figure 5:**
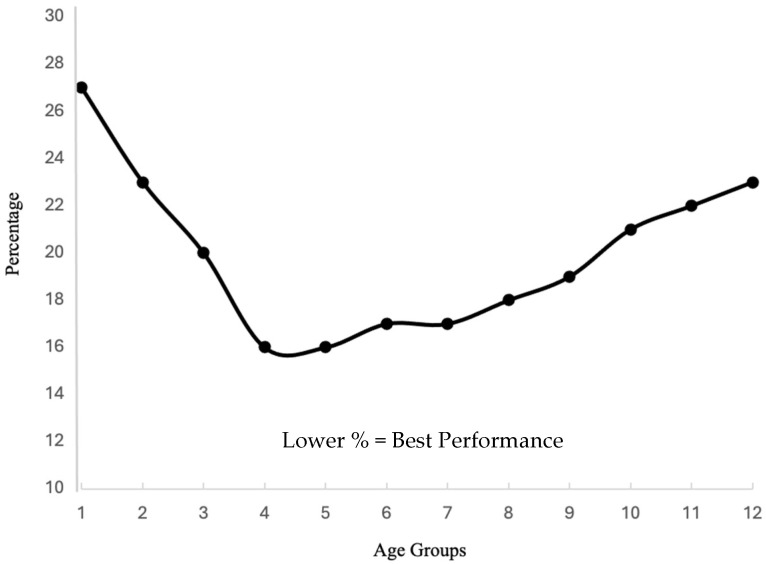
Example of the inverted U as seen in the latent smooth pursuit percentage demonstrating eye movement behavior across the lifespan.

Interestingly, a pattern is revealed when reviewing individual variables throughout Table A8, Table A9, Table A10, Table A11, Table A12, Table A13 and Table A14, showing an inverted bell curve in the results. For example, latent smooth pursuit (Table A8, Figure 5) is higher (worse) among the young (6- and 7-year-olds with a median of 26.57%). Then, there was a consistent reduction in percentage, showing an improvement in eye movement behavior until middle adulthood (Group 6: 32–38-year-olds). This is followed by a consistent increase in latent smooth pursuit percentages from middle age to elderly (Group 12: 77–80-year-olds) showing similar results to the very young (Group 1, 6-to-7-year-olds, and Group 2, 8–11-year-olds).

This inverted bell curve reveals a change toward improvement in eye movement behavior until ones 30 s, then a gradual, consistent, and significant decline until 80 years of age. The decline is at a more gradual rate than the improvement seen in early years. Furthermore, the decline does not reach the initial, early age levels of high latent smooth pursuit percentages, indicating poor performance. This trend occurs not only in smooth pursuit eye movement metrics but also in saccades, fixations, and vergence-related variables (e.g., Table A12: On Target). Sensitive measures of variance and efficiency also follow the same pattern (see Table A12). These findings support other lifespan developmental research indicating changes early and late in life, with peak abilities stabilizing in early adulthood [20,21,22].

Eye movement speed is slowest in Groups 1 and 12, the young and elderly (Table A11, saccadic amplitude, 188 and 191 degrees, respectively). However, when viewed in conjunction with saccadic targeting, we see that the elderly are slower and more accurate than the young (9.18 in Group 1 and 11.05 in Group 12). This is further validated when viewing the results of the speed–accuracy trade-off (Table A11), whereby there is a reduction in speed in both Groups 1 and 12 but a higher accuracy even with the speed reduction for Group 12, indicating a trade-off that reveals slower speed with greater accuracy for the elderly population (3.60). In contrast, in middle age, people can be both fast and accurate with their eye movements, as demonstrated by Group 5, 26-to-31-year-olds, whose speed–accuracy trade-off was almost double that of the young and the elderly (5.67). These results are consistent with research that shows older adults are slower than younger adults in completing most tasks [22]. Some reasons for this may include older adults being reluctant to commit errors and unwilling to adopt a “fast-and-careless” attitude.

These results may be useful in future eye movement studies where people of different ages participate. Adjustments to the design of research, specifically the methodologies and related results, should carefully consider the impact of age. Studies may consider grouping participants by age or only including specific age ranges, should they wish age, not to be a confounding variable.

This model holds many advantages over models utilized in the past. Its ability to accurately differentiate by age groups solely based on eye movement data sets it apart. This differentiation has allowed for a more thorough identification of developmental biomarkers. The work and findings are predicated on a large sample size, thereby enhancing the generalizability of the findings.

While this study marks the potential for updating our understanding of age-related changes in eye movement patterns, certain limitations exist. Specifically, there are limitations in its use of cross-sectional cohorts [23]. Although this study attempts to mitigate this limitation by having a large sample size, future research should consider longitudinal tracking to assess possible generational differences that could serve as confounders (e.g., technology usage). An examination of the data may further stratify persons into age–gender cohorts. On a related note, a limitation does, indeed, exist, in that we had more female participants than male participants. This introduces the potential for bias predicated on gender, a limitation future work should aim to address. Finally, charting typical developmental trajectories in each type of eye movement (fixations, saccades, pursuits, and vergence) may further stratify groups and, in turn, may more precisely assist clinicians and researchers in defining eye movements across the human lifespan.

Eye movements, measured using eye trackers, provide quantifiable reflections of eye movement behavior that may, in turn, be used to group persons based on age. Such information may be used as a digital biomarker. Digital biomarkers are objective measures that capture the state of a cell or, in this case, the eye movements of a human being [24]. Digital biomarkers are collected via computing systems such as digital services, wearable technology, or computer technologies that can explain, influence, or predict health-related outcomes [25].

Digital biomarkers can play an important role in uncovering a person’s health and wellness for early disease detection, enabling healthcare providers to administer early and targeted treatments that may slow, reduce, or even cure disease in patients [26]. As such, it is vital to be able to compare expected (normative) controls with potential disease states [27,28]. Similarly, the biomarkers identified have implications for clinicians in practice, as these biomarkers could allow for detecting various disorders that would be otherwise difficult or delayed without quantifiable metrics. In this regard, these metrics allow for establishing benchmarks that are fluid with age instead of uniform across the lifespan. For example, the inverted bell curve illuminated in this work subsequently enables professionals to adjust patient expectations, thereby enhancing individualized care. As it pertains to assistive technology, clinicians can and should consider facets such as the speed–accuracy trade-off to inform both its development and adjustment to meet the needs of populations for which these devices are meant to support. These data can be used to establish baseline measures and monitor the patient over time. As eye movements significantly change over the lifespan, these normative comparisons for biomarker conclusions must be accurately determined using the appropriate age-related considerations. When biomarkers are evaluated within the context of demographic factors, the results may provide clinicians with important, previously unknown information that can aid clinical decision-making [29].

The advancement of medical science has brought remarkable improvements in the diagnosis and treatment of diseases. However, many diagnostic techniques remain invasive, often causing patients discomfort, risk, and potential complications. This study has identified eye movements as noninvasive biomarkers of human function correlated with age that are easy to obtain and quantify. There are high correlations between pathologies of eye movement and a host of neurological disorders [1]. Eye movement biomarkers have great promise to revolutionize disease detection, monitoring, and treatment, enhancing patient care and outcomes. Developing noninvasive disease biomarkers with untold potential benefits, current advancements, and future implications is essential.

Many diseases, particularly early-stage diseases, might not be easily accessible or identifiable. Noninvasive biomarkers such as eye movements present an exciting purpose to mitigate these issues as further clinical research develops a greater understanding of human function in health and disease. The statistically different performance of eye movements throughout the life span provides baseline measurements of function that might be compared to each individual. Deviations from expected baseline performance promote a deeper investigation of other human functions and systems integrity to identify disease or functional pathology before it becomes clinically evident.

Early detection of pathology can frequently result in an improved prognosis. Eye movement deviation from the baselines we have quantified can facilitate the early detection of diseases crucial for conditions like cancer, cardiovascular diseases, and neurodegenerative disorders. An early diagnosis often leads to better treatment outcomes and improved survival rates. Patients can be expected to be more comfortable with biomarker identification through noninvasive technology instead of invasive testing, as described in our investigation. This reduction in discomfort can lead to higher compliance rates for regular monitoring and follow-up, which is essential for managing chronic diseases and monitoring treatment efficacy.

A cost-effective consequence of utilizing eye tracking as a noninvasive diagnostic method can be realized by requiring fewer resources and infrastructure than invasive techniques. This cost reduction can make healthcare more accessible, particularly in resource-limited settings. Eye tracking technology allows for frequent and real-time monitoring of disease progression and treatment response monitoring, enabling better control and management of various medical conditions correlated with brain function and volitional eye movements.

Significant progress has been made in identifying and validating correlations between eye movement, brain function, and various clinical syndromes. This investigation quantifies eye movement by age groups with sample size and power necessary to establish biomarkers across the life span, increasing the value of eye movement as a noninvasive biomarker. The continued development of noninvasive biomarkers, such as those identified in this investigation, holds immense promise for the future of medicine. As research progresses, identifying novel biomarkers and refining detection technologies will likely lead to more accurate, efficient, and patient-friendly diagnostic methods. Moreover, noninvasive biomarkers will significantly benefit the desire to establish personalized medicine. These biomarkers can provide detailed insights into an individual’s unique health profile, enabling tailored treatment strategies that improve outcomes and reduce adverse effects. 

Developing noninvasive biomarkers is a crucial advancement in modern medicine, offering significant benefits over traditional invasive diagnostic methods. Eye movements as noninvasive biomarkers can transform disease diagnosis and management by facilitating early detection, improving patient comfort, reducing costs, and enabling real-time monitoring. Continued research and innovation in this field will undoubtedly lead to improved health outcomes and a higher quality of life for patients worldwide. As we move forward, integrating these biomarkers into clinical practice will be essential in realizing their full potential and advancing healthcare for all.

This investigation of eye movements across the human lifespan has generated significant new knowledge, advancing our understanding of how eye movements change with age. We have identified developmental biomarkers that can be used in clinical and research settings. The primary breakthrough of this research lies in its ability to accurately categorize individuals into distinct age groups based solely on eye movement data. This was achieved with remarkable accuracy by studying a vast age range with a large sample size, ensuring the robustness and generalizability of our findings.

One of the key findings in our work is the identification of age-specific changes in eye movement patterns. This investigation revealed that the youngest and oldest age groups exhibit more rapid changes in eye movements, resulting in narrower age spans for these groups. This suggests that early development and late-life decline are periods of heightened sensitivity to changes in eye movement behavior.

Our study uncovered complex patterns in eye movement behaviors that vary significantly with age. The analysis demonstrated a unique pattern: eye movement efficiency improves until the early 30s and then gradually declines. This pattern was consistent across various types of eye movements, including fixations, saccades, pursuits, and vergence. For instance, latent smooth pursuit metrics showed higher (worse) values in young children, a consistent reduction (improvement) in early adulthood, and then a gradual increase (worsening) in older age.

Additionally, the speed–accuracy trade-off revealed further complexity in eye movement patterns. While the youngest and oldest groups exhibited slower eye movement speeds, elderly individuals demonstrated higher accuracy, suggesting a prioritization of accuracy over speed. This finding indicates that older adults may adopt more cautious eye movement strategies and are likely to avoid errors, which aligns with broader research on age-related changes in cognitive and motor functions.

The new stratification of eye movement function by age developed from this research has the potential to significantly upgrade assistive technologies and healthcare stakeholders’ decision-making. Accurately differentiating age groups based on eye movement data can enhance the development of personalized assistive technologies. For example, eye-tracking devices can be tailored for different age groups’ specific needs and capabilities, improving usability and effectiveness for young and elderly individuals.

In healthcare, identifying age-related eye movement biomarkers can aid in early detection and monitoring of neurological and cognitive disorders. Clinicians can use these biomarkers to establish patient baseline measures and track changes, allowing for timely interventions. For instance, deviations from normative eye movement patterns could signal early stages of conditions such as Parkinson’s disease or dementia, enabling healthcare providers to administer targeted treatments that may slow or mitigate disease progression.

Moreover, understanding the nuances of eye movement patterns across the lifespan can inform the design of clinical studies and trials. Researchers can group participants more accurately based on age-related eye movement data, reducing the potential for age to confound study results. This precision can lead to more reliable and valid findings, ultimately enhancing the quality of research on eye movements and cognitive health.

Our investigation has produced substantial new knowledge by elucidating how eye movements change across the human lifespan while identifying developmental biomarkers associated with these changes. The discovery of complex patterns in eye movement behavior, such as the inverted bell curve and the speed–accuracy trade-off, adds depth to our understanding of the factors affecting eye movements. The functional performance identified from this research holds significant promise for upgrading assistive technologies and informing healthcare decision-making. Eye movement biomarkers can improve patient outcomes and advance developmental and clinical research by enabling early detection and personalized interventions.

One of this research’s primary breakthroughs is its remarkable ability to accurately categorize individuals into distinct age groups based solely on eye movement data. This innovative approach leverages the power of semi-supervised machine learning to analyze and interpret complex patterns in eye movements, yielding a high accuracy rate of 94.67%. Such precision enhances our understanding of developmental biomarkers and opens new avenues for practical applications in healthcare and technology.

Eye movements are a window into the brain’s functioning and reflect many cognitive processes. The four major types of eye movements—fixations, saccades, pursuits, and vergence—are influenced by age-related changes in neural and ocular systems. By capturing and analyzing these movements, researchers and clinicians can identify subtle variations correlating with different stages of human development and aging. This study collected eye movement data from a large and diverse sample of 46,655 participants ranging from 6 to 80 years old. This extensive dataset enabled the machine learning model to detect and learn the unique characteristics of eye movements associated with each age group. This granular analysis revealed patterns that are not easily discernible through traditional observational methods, thus highlighting the sophistication and capability of machine learning in biomedical research.

This study’s semi-supervised machine learning model is designed to handle vast numbers of data, identify patterns, and make accurate predictions. It combines elements of supervised learning, where the model is trained on a labeled dataset, with unsupervised learning, where it identifies hidden patterns in unlabeled data. This hybrid approach enhances the model’s ability to generalize from the data and improves accuracy.

By inputting eye movement data into the model, we stratified individuals into 12 distinct age groups based on intricate details of eye movement behavior, such as the frequency and duration of fixations, the amplitude and speed of saccades, the smoothness of pursuit movements, and the coordination of vergence. These parameters vary systematically with age, reflecting developmental and degenerative changes in the brain and visual system.

The ability to categorize individuals into age groups based on eye movement data has profound implications for identifying developmental biomarkers. Developmental biomarkers are measurable indicators that reflect the human body’s average growth and aging processes. In this context, eye movement patterns serve as biomarkers that can track cognitive and neural development progression across the lifespan. The breakthrough in accurately categorizing age groups based on eye movement data has significant practical applications. In healthcare, this capability can enhance the early detection and monitoring of neurological and cognitive disorders. Clinicians can use age-specific eye movement biomarkers to identify deviations from typical development, enabling timely interventions. For example, abnormalities in eye movement patterns could indicate early stages of neurodegenerative diseases like Parkinson’s or Alzheimer’s, allowing for earlier diagnosis and targeted treatment.

Using assistive technologies, this research can inform the development of personalized eye-tracking devices. These devices can be tailored to accommodate different age group’s specific needs and capabilities, improving usability and effectiveness for young children and elderly individuals. By incorporating age-related variations in eye movement behavior, these technologies can provide users with more accurate and reliable support. Furthermore, the insights gained from this study can improve the design of clinical trials and research studies. By grouping participants based on precise age-related eye movement data, researchers can reduce the potential for age to confound study results. This precision enhances the validity and reliability of research findings, ultimately advancing the field of developmental and clinical neuroscience.

Accurately categorizing individuals into distinct age groups based solely on eye movement data represents a significant breakthrough in biomedical research. This innovative approach leverages the power of machine learning to uncover intricate patterns in eye movements, providing valuable insights into developmental biomarkers. The practical applications of this research are vast, ranging from early detection and monitoring of neurological disorders to the development of personalized assistive technologies. This study paves the way for improved healthcare outcomes and technological innovations by advancing our understanding of age-related changes in eye movement behavior.

Age-related diseases manifest at different stages of life, each with unique challenges and implications. Understanding the progression and characteristics of these diseases from childhood to old age is crucial for effective diagnosis, treatment, and management. This investigation explores the spectrum of age-related eye movement performance. It promotes applications to health conditions that affect individuals at various life stages while allowing for discussion of their impact on health and quality of life.

During childhood, age-related diseases often stem from congenital and developmental origins. These conditions can significantly impact physical, cognitive, and emotional development, requiring early intervention to improve long-term outcomes. Eye movement biomarkers can document whether a child has obtained a similar performance capability as others of the same age. These biomarkers also serve to assist in the assessment of the success or failure of a variety of treatment interventions.

Age-related diseases span the entire human lifespan, from congenital and developmental disorders in childhood to chronic and neurodegenerative conditions in old age. Each stage of life presents unique challenges and opportunities for intervention. Understanding the progression and impact of these diseases is crucial for developing effective prevention, diagnosis, and treatment strategies. Identifying and quantifying eye movement performance by age has been central to this investigation. By addressing the specific needs of individuals at different life stages, healthcare providers can improve outcomes and enhance the quality of life for patients across the lifespan. Continued research and innovation are essential to tackle the evolving landscape of age-related diseases and to ensure optimal care for all individuals.

The continued development of eye movements as noninvasive biomarkers holds immense promise for the future of medicine. Identifying novel biomarkers and refining detection technologies will likely lead to even more accurate, efficient, and patient-centered diagnostic methods as research progresses. Developing noninvasive biomarkers is a crucial advancement in modern medicine, offering significant benefits over traditional invasive diagnostic methods. Eye movement quantification fits this developmental challenge well. Noninvasive eye movement biomarkers can potentially transform disease diagnosis and management by facilitating early detection, improving patient comfort, reducing costs, and enabling real-time monitoring. Continued research and innovation in this field will undoubtedly lead to improved health outcomes and a higher quality of life for patients worldwide. As we move forward, integrating these eye movement biomarkers into clinical practice will be essential in realizing their full potential and advancing healthcare for all.

## 5. Conclusions

In conclusion, this study aimed to benchmark age-based developmental biomarkers that can be used as milestones for clinicians and researchers to inform decision-making on patients’ health and wellness. The work revealed that machine learning could differentiate individuals into age groups predicated on eye movements. This differentiation was achieved with high accuracy. Additionally, patterns regarding biomarkers across age groups were illuminated. More rapid changes were seen in children and elderly individuals. At the same time, very few changes were observed in young adults. Middle-aged individuals experienced changes every five to six years. This work revealed an inverse bell curve. In this regard, across multiple eye movement types, children and elderly individuals had worse scores than young adults and middle-aged individuals. There was also a notable speed–accuracy trade-off among elderly individuals, with this group experiencing low speed but high accuracy.

It should be noted that children, too, have eye movement at a slower speed than other age groups. The application of this research in a clinical setting should be explored to assess its potential to detect disorders (e.g., Parkinson’s). Effective implementation and application in the real world could enhance the ecological validity of this work’s findings. It is the hope that this study and its results can be added to the toolkit for developmentalists and others who seek to evaluate and understand eye movement and associated neural substrates in typical (and, by default, atypical) development.

## Figures and Tables

**Table 1:** Age data for each age group defined by the model.

	Participants	Age Range (years)	Median Age	IQR Lower	IQR Upper	Male %	Female %
1	414	6 to 7	7	7	7	20.77	79.23
2	4299	8 to 11	10	9	11	25.05	74.95
3	4446	12 to 14	13	12	14	28.61	71.39
4	13,561	15 to 25	19	17	22	30.57	69.43
5	4716	26 to 31	28	27	30	27.14	72.86
6	3213	32 to 38	34	33	35	22.13	77.87
7	5225	39 to 45	41	39	43	21.88	78.12
8	3877	46 to 53	49	47	51	22.18	77.82
9	2522	54 to 60	57	55	59	21.37	78.63
10	2115	61 to 68	64	62	66	21.37	78.63
11	1077	69 to 76	72	70	74	23.86	76.14
12	231	77 to 80	78	77	79	21.21	78.79

**Table 2:** Multiple analysis of variance outcomes for the self-training classifier model.

Test	Wilks’ Lambda	F	*p*-Value
Circular Smooth Pursuit	0.863	36.206	<0.001
Horizontal Smooth Pursuit	0.857	35.966	<0.001
Vertical Smooth Pursuit	0.809	74.264	<0.001
Horizontal Saccades	0.796	68.545	<0.001
Vertical Saccades	0.791	82.525	<0.001
Fixation Stability	0.844	54.863	<0.001

## Data Availability

The data are not publicly available due to the expense and volume of cloud-based processes.

## Appendix A

**Table A1:** Circular smooth pursuit task, eye movement type, eye measured, variable name, and definition.

Eye Type	Variable Name	Eye	Algorithm Definition
Saccade	Saccade Percentage	Both	Eye movements greater than 30 degrees per second and calculated as a percentage of test time.
Fixation	Fixation Percentage	Both	A stopping point within 0.25 degree of dispersion for ≥100 ms. Reported as a percentage of the test time.
Pursuit	Horizontal Synchronization	Both	How far off on the X plane (coordinate) the user’s eyes were during the test. Perfect synchronization is a score of 1.0.
Pursuit	Vertical Synchronization	Both	How far off on the Y plane (coordinate) the user’s eyes were during the test. Perfect synchronization is a score of 1.0.
Pursuit	Predictive Smooth Pursuit	Both	The location of the user’s eyes within a velocity range of the target and positioned ahead or in-front-of the stimuli between 2 and 5 cm and reported as a percentage.
Pursuit	Latent Smooth Pursuit	Both	The location of the user’s eyes within a velocity range of the target and positioned behind the stimuli between 2 and 5 cm and reported as a percentage.
Pursuit	Smooth Pursuit Efficiency	Both	The error in the users’ gaze is from the ideal pathway. Lower is better, measured in millimeters.
Pursuit	Smooth Pursuit Variance	Both	The average variance from the ideal pathway from three segments of the pathway, middle, left/right, or up/down, measured in millimeters.
Vergence	Disassociated Phoria	Both	The deviation of the line of sight inward (eso +) or outward (exo −).
Pursuit	Gaze Radius Difference 2	Both	The differences in the size of gaze plots, between left and right eye at 67.5 degrees, measured in millimeters.
Pursuit	Gaze Radius Difference 3	Both	The differences in the size of gaze plots, between left and right eye at 112.5 degrees, measured in millimeters.
Pursuit	Gaze Radius Difference 4	Both	The differences in the size of gaze plots, between left and right eye at 157.5 degrees, measured in millimeters.
Pursuit	Gaze Radius Difference 5	Both	The differences in the size of gaze plots, between left and right eye at 202.5 degrees, measured in millimeters.
Pursuit	Gaze Radius Difference 6	Both	The differences in the size of gaze plots, between left and right eye at 247.5 degrees, measured in millimeters.
Pursuit	Gaze Radius Difference 8	Both	The differences in the size of gaze plots, between left and right eye at 337.5 degrees, measured in millimeters.
Pursuit	Size Difference	Both	The differences in the size of gaze plots, between left and right eye, measured in millimeters.
Pursuit	Shape Difference	Both	The deviation of gaze plot from its ideal circular shape, measured in millimeters.

**Table A2:** Horizontal smooth pursuit task, eye movement type, eye measured, variable name, and definition.

Eye Type	Variable Name	Eye	Algorithm Definition
Pursuit	Pathway Length Difference Left Side	Both	The average difference in distance between the right and left eye gaze pathways on the left eccentric gaze position. Ideal score is zero. Lower is better, measured in millimeters.
Pursuit	Pathway Length Difference Right Side	Both	The average difference in distance between the right and left eye gaze pathways on the right eccentric gaze position. Ideal score is zero. Lower is better, measured in millimeters.
Blink	Blink Rate	Both	The number of blinks over the total test time (frequency of blinks).
Blink	Blink Number	Both	The total tally of blinks
Pursuit	Smooth Pursuit Percentage	Both	Eye movements that follow the target within 30 degrees per second target and are reported as a percentage of the test time.
Saccade	Saccade Percentage	Both	Eye movements greater than 30 degrees per second and calculated as a percentage of test time.
Fixation	Fixation Percentage	Both	A stopping point within 0.25 degree of dispersion for ≥ 100 ms. Reported as a percentage of the test time.
Saccade	Saccade Number	Both	The total tally of saccades recorded throughout the test.
Pursuit	Smooth Pursuit Gain	Both	Eye movements that are ahead of the target that fall within a 4.36 degrees error radius
Saccade	Saccade Percentage Left side	Both	The number of saccades on the left portion of the screen reported as a percentage of test time.
Pursuit	Horizontal Synchronization	Both	How far from the X plane (coordinate) the user’s eyes were during the test. Perfect synchronization is a score of 1.0.
Vergence	Disassociated Phoria	Both	The deviation of the line of sight inward (eso +) or outward (exo −).
Pursuit	Eccentric Gaze Mean Left Side	Both	The mean distance between gaze points to the ideal path of stimuli in pursuit, on left side of the stimuli path (left 1/3rd)
Pursuit	Eccentric Gaze Mean Middle	Both	The mean distance between gaze points to the ideal path of stimuli in pursuit, in middle section of the stimuli path (middle 1/3rd)
Pursuit	Eccentric Gaze Mean Right Side	Both	The mean distance between gaze points to the ideal path of stimuli in pursuit, on right side of the stimuli path (right 1/3rd)
Pursuit	Efficiency	Both	The error in the gaze following a smooth pursuit stimulus.
Pursuit	Variance	Both	The average variance from the ideal pathway. We look at variance in three segments of the pathway, middle, left/right or up/down.
Pursuit	Smooth Pursuit Variance Difference	Both	The difference between the average variance from the ideal pathway, from three segments of the pathway, middle, left/right, or up/down.

**Table A3:** Vertical Smooth Pursuit Task, Eye Movement Type, Eye Measured, Variable Name, and Definition.

Eye Type	Variable Name	Eye	Algorithm Definition
Pursuit	Pathway Length Difference Top Side	Both	The average difference in distance between the right and left eye gaze pathways in the top side of the screen. Ideal score is zero. Lower is better, measured in millimeters.
Pursuit	Pathway Length Difference Bottom Side	Both	The average difference in distance between the right and left eye gaze pathways in the bottom side of the screen. Ideal score is zero. Lower is better, measured in millimeters.
Blink	Blink Rate	Both	The number of blinks over the total test time (frequency of blinks).
Saccade	Saccade Number	Both	The total tally of saccades recorded throughout the test.
Pursuit	Smooth Pursuit Gain	Both	Eye movements that are ahead of the target that fall within a 4.36 degree error radius
Pursuit	Vertical Synchronization	Both	How far from the Y plane (coordinate) the user’s eyes were during the test. Perfect synchronization is a score of 1.0.
Vergence	Disassociated Phoria	Both	The deviation of the line of sight inward (eso +) or outward (exo −).
Pursuit	Eccentric Gaze Variability Middle	Both	The variability in the middle portion of the screen that is outside the “box”. Lower is better, measured in millimeters.
Pursuit	Efficiency	Both	The error in the gaze following a smooth pursuit stimulus, measured in millimeters.
Pursuit	Variance	Both	The average variance from the ideal pathway. We look at the variance in three segments of the pathway, middle, left/right, or up/down. Measured in millimeters.
Pursuit	Eccentric Gaze Variability Bottom	Both	The variability on the bottom portion of the screen that is outside the “box.” Lower is better, measured in millimeters.
Pursuit	Eccentric Gaze Mean Middle Difference	Both	The mean distance between gaze points to the ideal path of stimuli in pursuit, in middle section of the stimuli path (middle 1/3rd)
Pursuit	Smooth Pursuit Variance Difference	Both	The difference in the average variance from the ideal pathway. We look at variance in three segments of the pathway, middle, left/right, or up/down.

**Table A4:** Horizontal saccades task, eye movement type, rye measured, variable name, and definition.

Eye Type	Variable Name	Eye	Algorithm Definition
Fixation	Fixation Number	Both	Stopping points or about-turn of the user’s gaze. Fixations are tallied throughout the test and reported as the total number of fixations.
Fixation	On Target	Both	On-Target refers to accuracy and proximity of eye gaze point to the dot when fixating. Measured in millimeters.
Fixation	Band 2 Under	Both	Total number of targets undershot by 9–18 mm.
Fixation	Band 3 Over	Both	Total number of targets overshot on the horizontal plane by 18–36 mm.
Fixation	Band 3 Under	Both	Total number of targets undershot on the horizontal plane by 18–36 mm.
Fixation	Missed	Both	No target is hit, and the user has passed the center of the screen in the direction of the target.
Fixation	Missed Under Right Side	Both	The user misses and hits before the right target. Miss is more than 36 mm.
Saccade	Saccadic Efficiency	Both	The average distance the users’ saccade is from the ideal pathway. Measured in millimeters.
Saccade	Saccadic Targeting	Both	The distance each “hit”, or fixation was compared to the ideal target.
Saccade	Saccadic Velocity	Both	The average velocity made by the saccades across the test time. Measured in degrees per second.
Saccade	Speed Accuracy Trade Off	Both	Speed divided by accuracy. Measured as degrees per second divided by millimeters.
Saccade	Saccadic Recovery	Both	The difference in the path taken before and after a fixation. A wide, looping path is inefficient. A narrow path is ideal. Measured in millimeters.
Saccade	Saccadic Amplitude	Both	Average distance between consecutive turning points. Measured in millimeters.
Saccade	Q Ratio	Both	The ratio of “Peak Velocity” to “Average Velocity” in the saccadic interval.

**Table A5:** Vertical saccades task, eye movement type, eye measured, variable name, and definition.

Eye Type	Variable Name	Eye	Algorithm Definition
Fixation	Fixation Number	Both	Stopping points or about-turn of the user’s gaze. Fixations are tallied throughout the test and reported as the total number of fixations.
Fixation	On Target	Both	On-Target refers to accuracy and proximity of eye gaze point to the dot when fixating. Measured in millimeters.
Fixation	Band 2 Over	Both	Total number of targets overshot by 9–18 mm.
Fixation	Band 2 Under	Both	Total number of targets undershot by 9–18 mm.
Fixation	Band 3 Under	Both	Total number of targets undershot by 18–36 mm.
Fixation	Missed	Both	No target is hit, and the user has passed the center of the screen in the direction of the target.
Saccade	Saccadic Efficiency	Both	The average distance the users’ saccade is from the ideal pathway. Measured in millimeters.
Saccade	Saccadic Targeting	Both	The distance each “hit”, or fixation was compared to the ideal target.
Saccade	Saccadic Velocity	Both	The average velocity made by the saccades across the test time. Measured in degrees per second.
Saccade	Saccadic Variance	Both	The spread or variability in the saccadic pathways.
Saccade	Saccadic Amplitude	Both	Average distance between consecutive turning points. Measured in millimeters.
Saccade	Q Ratio	Both	The ratio of “Peak Velocity” to “Average Velocity” in the saccadic interval.

**Table A6:** Fixation stability task, eye movement type, eye measured, variable name, and definition.

Eye Type	Variable Name	Eye	Algorithm Definition
Vergence	Depth	Both	The difference between the point of convergence and the screen. The ideal result is zero. Negative numbers show a point of convergence behind the screen. A positive number shows a point of convergence in front of the screen. Close to zero is best.
Vergence	Convergence Point	Both	The average distance between the “point of convergence of eyes” from the stimuli location on *z*-axis in a 3D plane.
Fixation	Bivariate Contour Ellipse Analysis (BCEA)	Both	Microsaccades and drifts of the human eye cause corrections of the eye back to a central point. These slight eye movements form an area of dispersion in an ellipse shape measured by the BCEA.
Fixation	Gaze Position 1	Left	The percentage of gaze that fell less than or equal to 1 degree of the target center from the left eye only.
Fixation	Gaze Position 1	Right	Percentage of gaze that fell less than or equal to 1 degree of the target center from the right eye only.
Fixation	Gaze Position 3	Left	The percentage of gaze that fell between 2 and 4 degrees from the target center from the left eye only.
Fixation	Gaze Position 3	Right	The percentage of gaze that fell between 2 and 4 degrees from the target center from the left eye only.
Fixation	Disassociated Tropia	Both	Deviation of the line of sight, where one eye is above the other. If it is negative value, then the left eye is higher than the right. If a positive value, then the right eye is higher than the left. Close to zero is best.
Fixation	Horizontal Targeting Displacement	Left	The displacement between target (FS stimuli) and the Mean of gaze points corresponding to that stimulus, on *X*-axis. Measured in millimeters.
Fixation	Horizontal Targeting Displacement	Right	The displacement between target (FS stimuli) and the Mean of gaze points corresponding to that stimulus, on *Y*-axis. Measured in millimeters.
Fixation	Vertical Targeting Displacement	Left	The displacement between target (FS stimuli) and the mean of gaze points corresponding to that stimulus, on the *Y*-axis. Measured in millimeters.
Fixation	Vertical Targeting Displacement	Right	The displacement between target (FS stimuli) and the mean of gaze points corresponding to that stimulus, on the *Y*-axis. Measured in millimeters.
Fixation	Fixation Dispersion	Right	The distance between each gaze point and target stimuli averaged over the entire test for all gaze points. Measured in millimeters.

**Table A7:** Pupil measures, calibration task, eye measured, variable name, and definition.

Eye Type	Variable Name	Eye	Definition
Pupil	Pupil Diameter Mean	Both	The average size of the pupil when the stimuli is centered on the screen.
Pupil	Pupil Diameter Difference	Both	The difference in size between the left and right pupils. Measured in millimeters.

## Appendix B

**Table A8:** Circular smooth pursuit median and interquartile range for the 17 contributing eye movement variables.

	**Saccade %**	**Fixation %**	**Horizontal**	**Vertical**	**Predictive Smooth**	**Latent Smooth**
			**Synchronization**	**Synchronization**	**Pursuit**	**Pursuit**
1	4.43 (2.75−6.43)	4.96 (4.07−5.82)	0.97 (0.96−0.97)	0.92 (0.90−0.94)	4.31 (2.23−7.23)	26.57 (20.50−33.02)
2	3.83 (2.28−5.83)	4.77 (4.01−5.59)	0.96 (0.96−0.97)	0.92 (0.90−0.94)	3.47 (1.51−6.53)	22.71 (16.77−29.13)
3	3.23 (1.78−5.36)	4.71 (3.97−5.42)	0.96 (0.96−0.97)	0.92 (0.91−0.94)	2.92 (1.22−5.70)	19.51 (13.71−25.64)
4	2.37 (1.24−4.27)	4.64 (3.94−5.34)	0.96 (0.96−0.97)	0.93 (0.91−0.94)	2.06 (0.78−4.45)	16.19 (10.96−22.57)
5	2.07 (1.12−3.83)	4.71 (4.03−5.39)	0.96 (0.96−0.97)	0.93 (0.91−0.94)	1.79 (0.66−4.14)	16.10 (10.68−22.42)
6	2.21 (1.15−4.01)	4.74 (4.05−5.43)	0.96 (0.96−0.97)	0.92 (0.91−0.94)	1.89 (0.72−4.17)	16.64 (11.12−22.87)
7	2.34 (1.22−4.21)	4.71 (4.03−5.41)	0.96 (0.96−0.97)	0.92 (0.90−0.94)	1.93 (0.70−4.41)	17.18 (11.61−23.99)
8	2.44 (1.30−4.34)	4.70 (4.02−5.41)	0.96 (0.96−0.97)	0.92 (0.90−0.94)	2.02 (0.73−4.76)	18.28 (12.32−25.78)
9	2.77 (1.45−4.90)	4.72 (3.99−5.42)	0.96 (0.96−0.97)	0.92 (0.90−0.94)	2.32 (0.89−5.33)	19.33 (12.98−26.53)
10	3.06 (1.61−5.33)	4.72 (3.98−5.41)	0.96 (0.96−0.97)	0.91 (0.89−0.93)	2.52 (0.94−5.39)	20.66 (14.14−28.49)
11	3.82 (2.11−6.02)	4.68 (3.96−5.43)	0.96 (0.96−0.97)	0.91 (0.89−0.93)	3.44 (1.35−7.22)	21.52 (15.13−28.66)
12	4.54 (2.56−6.87)	4.74 (3.81−5.46)	0.96 (0.96−0.97)	0.91 (0.89−0.93)	3.87 (1.68−6.52)	22.53 (16.07−30.67)
	**Smooth Pursuit**	**Smooth Pursuit**	**Disassociated**	**Gaze Radius**	**Gaze Radius**	**Gaze Radius**
	**Efficiency**	**Variance**	**Phoria**	**Difference 2**	**Difference 3**	**Difference 4**
1	14.02 (12.15−18.16)	13.41 (9.14−21.38)	−0.31 (−0.66−0.05)	47.37 (44.69−50.25)	46.56 (43.23−49.62)	46.51 (43.24−49.15)
2	12.72 (11.37−15.63)	13.47 (8.83−19.75)	−0.27 (−0.64−0.06)	48.35 (45.87−50.84)	46.75 (43.67−49.64)	47.13 (44.07−50.08)
3	11.83 (10.80−13.77)	12.46 (8.20−18.10)	−0.26 (−0.65−0.09)	49.16 (46.63−51.67)	47.10 (43.94−49.97)	47.53 (44.74−50.47)
4	11.15 (10.30−12.43)	12.49 (7.99−17.72)	−0.31 (−0.70−0.04)	49.22 (46.92−51.62)	47.42 (44.79−50.03)	47.82 (45.08−50.49)
5	11.13 (10.32−12.30)	12.66 (8.02−17.98)	−0.35 (−0.73−0.01)	49.11 (46.74−51.52)	47.65 (45.06−50.32)	47.66 (44.94−50.48)
6	11.25 (10.40−12.42)	13.03 (8.43−18.47)	−0.37 (−0.75−0.01)	49.37 (46.91−51.63)	47.79 (45.01−50.26)	47.92 (45.18−50.75)
7	11.39 (10.51−12.66)	13.14 (8.50−18.33)	−0.35 (−0.73−0.01)	49.20 (46.76−51.64)	47.54 (44.73−50.25)	47.84 (45.00−50.60)
8	11.63 (10.68−13.03)	13.62 (8.71−18.67)	−0.35 (−0.74−0.00)	49.09 (46.57−51.64)	47.41 (44.51−50.26)	47.92 (44.93−50.77)
9	11.81 (10.80−13.40)	13.28 (8.60−18.66)	−0.33 (−0.72−0.00)	49.09 (46.46−51.67)	47.57 (44.44−50.73)	47.95 (44.70−50.94)
10	12.13 (11.03−13.75)	13.09 (8.59−18.65)	−0.33 (−0.71−0.05)	49.10 (46.45−51.81)	47.21 (43.93−50.32)	48.20 (45.16−51.25)
11	12.64 (11.31−14.53)	13.99 (9.11−19.39)	−0.32 (−0.71−0.04)	48.43 (45.71−51.15)	47.27 (43.82−50.98)	47.81 (44.50−50.92)
12	12.74 (11.80−14.97)	13.83 (10.11−19.53)	−0.34 (−0.81−0.05)	48.11 (45.42−51.95)	46.65 (43.10−50.38)	48.70 (45.05−51.57)
	**Gaze Radius**	**Gaze Radius**	**Gaze Radius**	**Size Difference**	**Shape Difference**	
	**Difference 5**	**Difference 6**	**Difference 8**			
1	47.15 (44.82−49.96)	47.50 (44.23−50.19)	47.32 (45.07−49.80)	47.42 (45.92−48.73)	4.56 (3.46−6.32)	
2	47.12 (43.97−49.78)	47.96 (45.06−50.56)	48.11 (45.49−50.39)	47.80 (46.28−49.01)	4.39 (3.44−5.90)	
3	46.93 (44.00−49.62)	48.00 (45.09−50.54)	48.39 (46.03−50.73)	48.15 (46.83−49.28)	4.30 (3.41−5.68)	
4	47.02 (44.33−49.60)	47.79 (45.22−50.26)	48.42 (46.11−50.68)	48.27 (47.17−49.27)	4.11 (3.24−5.25)	
5	47.12 (44.44−49.54)	47.78 (45.13−50.40)	48.33 (45.86−50.53)	48.26 (47.19−49.28)	4.07 (3.23−5.22)	
6	47.15 (44.48−49.67)	47.89 (45.35−50.38)	48.34 (46.08−50.59)	48.33 (47.26−49.35)	4.11 (3.25−5.33)	
7	47.07 (44.39−49.69)	48.03 (45.45−50.71)	48.21 (45.90−50.50)	48.23 (47.13−49.31)	4.15 (3.27−5.35)	
8	47.01 (44.24−49.84)	48.28 (45.52−50.99)	48.10 (45.60−50.41)	48.21 (47.04−49.37)	4.26 (3.35−5.47)	
9	47.16 (44.43−50.14)	48.58 (45.56−51.25)	48.19 (45.61−50.61)	48.26 (47.03−49.50)	4.46 (3.50−5.76)	
10	47.07 (44.08−49.89)	48.67 (45.66−51.77)	48.01 (45.39−50.61)	48.25 (46.98−49.42)	4.47 (3.56−5.77)	
11	47.24 (43.38−50.22)	48.50 (45.59−52.09)	47.93 (44.70−50.28)	48.08 (46.56−49.38)	4.70 (3.66−6.23)	
12	46.98 (43.15−50.23)	49.03 (45.01−52.30)	47.35 (43.68−50.10)	47.90 (46.50−49.33)	5.23 (4.07−6.45)	

**Table A9:** Horizontal smooth pursuit median and interquartile range for the 18 contributing eye movement variables.

	**Pathway Length**	**Pathway Length**	**Blink Rate**	**Blink Number**	**Smooth Pursuit %**	**Saccade %**
	**Difference (L)**	**Difference (R)**				
1	0.31 (−2.27−2.88)	−1.15 (−5.31−1.60)	0.00 (0.00−0.01)	2.00 (0.00−3.00)	92.05 (89.12−94.29)	2.52 (1.20−4.49)
2	0.63 (−1.57−3.63)	−1.08 (−4.47−2.06)	0.01 (0.00−0.01)	2.00 (0.00−3.00)	92.55 (89.67−94.84)	2.16 (0.95−4.17)
3	0.76 (−1.52−3.93)	−1.10 (−4.32−2.11)	0.01 (0.00−0.01)	1.00 (0.00−3.00)	93.17 (90.42−95.31)	1.82 (0.88−3.71)
4	1.19 (−1.08−4.47)	−1.41 (−4.73−1.70)	0.01 (0.00−0.02)	1.00 (0.00−3.00)	93.71 (91.21−95.78)	1.38 (0.81−3.02)
5	1.46 (−0.80−4.84)	−2.08 (−5.44−1.15)	0.01 (0.00−0.01)	2.00 (1.00−4.00)	93.70 (91.24−95.68)	1.37 (0.87−2.91)
6	1.43 (−0.87−4.54)	−1.86 (−5.16−1.22)	0.01 (0.00−0.01)	2.00 (1.00−4.00)	93.59 (91.05−95.74)	1.39 (0.76−3.05)
7	1.33 (−0.77−4.54)	−2.30 (−5.48−0.81)	0.01 (0.00−0.01)	2.00 (1.00−4.00)	93.44 (90.88−95.56)	1.45 (0.81−3.08)
8	1.29 (−0.96−4.46)	−2.51 (−5.79−0.60)	0.01 (0.00−0.01)	2.00 (1.00−4.00)	93.46 (90.86−95.66)	1.45 (0.62−3.20)
9	1.16 (−1.28−4.26)	−2.60 (−5.91−0.48)	0.01 (0.00−0.01)	2.00 (1.00−4.00)	93.44 (90.60−95.72)	1.48 (0.46−3.21)
10	0.88 (−1.53−3.74)	−2.45 (−5.62−0.65)	0.01 (0.00−0.01)	2.00 (1.00−4.00)	93.55 (90.62−95.72)	1.43 (0.48−3.19)
11	0.58 (−1.62−3.60)	−2.60 (−6.05−1.09)	0.01 (0.00−0.01)	2.00 (1.00−4.00)	93.07 (90.20−95.47)	1.63 (0.53−3.41)
12	0.62 (−1.83−3.23)	−2.77 (−5.58−1.28)	0.00 (0.00−0.01)	2.00 (1.00−4.00)	93.54 (90.48−95.91)	1.80 (0.44−3.66)
	**Fixation %**	**Saccade Number**	**Smooth Pursuit Gain**	**Saccade % (L)**	**Horizontal**	**Disassociated**
					**Synchronization**	**Phoria**
1	5.10 (3.37−6.64)	4.00 (3.00−6.00)	10.91 (5.69−17.12)	1.09 (0.17−2.29)	0.89 (0.86−0.91)	0.45 (0.18−0.78)
2	4.83 (3.25−6.71)	4.00 (2.00−6.00)	11.38 (6.16−17.98)	0.90 (0.15−2.09)	0.89 (0.86−0.92)	0.44 (0.20−0.82)
3	4.65 (3.14−6.46)	3.00 (2.00−5.00)	11.43 (5.80−19.28)	0.72 (0.12−1.77)	0.89 (0.86−0.91)	0.45 (0.21−0.82)
4	4.26 (2.39−6.13)	2.00 (1.00−4.00)	9.92 (4.45−17.94)	0.45 (0.00−1.37)	0.88 (0.86−0.91)	0.48 (0.22−0.89)
5	4.33 (2.84−6.22)	2.00 (1.00−4.00)	9.04 (3.76−17.07)	0.47 (0.00−1.37)	0.88 (0.86−0.91)	0.51 (0.25−0.95)
6	4.29 (2.99−6.43)	2.00 (1.00−4.00)	9.77 (4.21−17.89)	0.45 (0.00−1.43)	0.88 (0.86−0.91)	0.53 (0.25−0.93)
7	4.68 (3.11−6.46)	3.00 (1.00−5.00)	10.07 (4.57−18.30)	0.50 (0.00−1.51)	0.88 (0.86−0.91)	0.52 (0.23−0.92)
8	4.42 (2.73−6.55)	3.00 (1.00−5.00)	11.66 (5.04−20.93)	0.51 (0.00−1.57)	0.88 (0.86−0.91)	0.52 (0.25−0.90)
9	4.48 (2.66−6.47)	3.00 (1.00−5.00)	12.54 (5.86−22.31)	0.56 (0.13−1.62)	0.88 (0.86−0.91)	0.51 (0.24−0.87)
10	4.45 (2.92−6.53)	3.00 (1.00−5.00)	13.46 (6.31−23.48)	0.49 (0.00−1.56)	0.88 (0.86−0.91)	0.50 (0.22−0.84)
11	4.77 (3.01−6.77)	3.00 (2.00−5.00)	16.27 (9.03−25.13)	0.67 (0.14−1.65)	0.88 (0.85−0.91)	0.48 (0.23−0.85)
12	4.05 (2.43−6.17)	3.00 (2.00−6.00)	19.13 (9.18−27.35)	0.84 (0.14−1.77)	0.88 (0.85−0.91)	0.49 (0.21−0.86)
	**Efficiency**	**Variance**	**Smooth Pursuit**	**Eccentric Gaze**	**Eccentric Gaze**	**Eccentric Gaze**
			**Variance Difference**	**Mean Left Side**	**Mean Middle**	**Mean Right Side**
1	11.59 (7.84−20.24)	12.35 (7.23−16.77)	1.09 (0.48−2.43)	1.03 (−0.88−2.82)	0.66 (−1.02−2.38)	0.80 (−0.98−2.69)
2	9.45 (7.28−15.96)	12.40 (8.01−17.22)	0.92 (0.39−2.12)	0.61 (−1.32−2.47)	0.25 (−1.37−1.96)	0.63 (−1.26−2.46)
3	8.29 (6.77−12.66)	12.07 (7.73−16.60)	0.84 (0.37−2.01)	0.52 (−1.47−2.49)	0.12 (−1.54−1.90)	0.47 (−1.41−2.28)
4	7.32 (6.32−9.51)	11.81 (7.77−16.08)	0.90 (0.36−2.12)	0.48 (−1.46−2.52)	0.14 (−1.58−1.88)	0.36 (−1.54−2.26)
5	7.25 (6.33−8.92)	11.95 (8.12−16.52)	0.92 (0.37−2.09)	0.60 (−1.33−2.72)	0.15 (−1.64−1.94)	0.38 (−1.50−2.38)
6	7.33 (6.40−9.46)	12.36 (8.29−16.66)	0.98 (0.39−2.21)	0.66 (−1.25−2.72)	0.06 (−1.66−1.82)	0.37 (−1.53−2.45)
7	7.48 (6.45−9.76)	12.51 (8.29−16.86)	0.95 (0.38−2.42)	0.93 (−1.14−3.02)	−0.07 (−1.79−1.81)	0.40 (−1.53−2.39)
8	7.55 (6.48−9.99)	12.63 (8.68−16.81)	1.06 (0.44−2.68)	1.24 (−0.96−3.39)	−0.26 (−2.13−1.77)	0.37 (−1.70−2.40)
9	7.61 (6.57−10.04)	12.40 (8.38−16.63)	1.19 (0.46−3.04)	1.35 (−0.99−3.58)	−0.34 (−2.22−1.73)	0.50 (−1.72−2.61)
10	7.78 (6.62−10.22)	12.35 (8.30−16.62)	1.26 (0.47−3.26)	1.40 (−0.68−3.69)	−0.42 (−2.48−1.65)	0.38 (−1.84−2.79)
11	8.13 (6.90−11.25)	12.48 (8.29−16.89)	1.22 (0.49−3.49)	1.83 (−0.33−4.29)	−0.07 (−2.12−1.95)	0.65 (−1.58−2.95)
12	8.68 (7.11−12.38)	13.26 (8.43−16.71)	1.38 (0.49−3.32)	2.12 (−0.37−4.91)	−0.28 (−2.43−1.86)	−0.07 (−2.18−2.55)

**Table A10:** Vertical smooth pursuit median and interquartile range for the 13 contributing eye movement variables.

	**Pathway Length**	**Pathway Length**	**Blink Rate**	**Saccade Number**	**Smooth Pursuit**	**Vertical**	**Disassociated**
	**Difference (Top)**	**Difference (Bottom)**			**Gain**	**Synchronization**	**Phoria**
1	−0.41 (−3.49−1.80)	0.83 (−2.70−4.45)	0.01 (0.00−0.01)	8.00 (6.00−11.00)	22.67 (15.68−30.16)	0.78 (0.73−0.82)	0.49 (0.21−0.81)
2	0.03 (−2.70−2.72)	0.67 (−3.13−4.43)	0.01 (0.00−0.01)	8.00 (5.00−11.00)	23.97 (16.78−31.83)	0.76 (0.72−0.80)	0.49 (0.23−0.90)
3	0.14 (−2.55−2.97)	0.54 (−3.32−4.42)	0.01 (0.00−0.01)	7.00 (4.00−10.00)	26.01 (17.15−34.89)	0.75 (0.71−0.78)	0.51 (0.23−0.91)
4	0.17 (−2.52−2.91)	0.35 (−3.29−4.03)	0.01 (0.00−0.02)	5.00 (2.00−8.00)	24.86 (15.78−35.35)	0.74 (0.70−0.77)	0.53 (0.24−0.98)
5	0.13 (−2.46−3.03)	0.60 (−2.90−4.10)	0.01 (0.00−0.01)	4.00 (2.00−7.00)	24.29 (14.09−35.16)	0.73 (0.70−0.77)	0.57 (0.27−1.04)
6	0.02 (−2.68−2.90)	0.59 (−2.83−4.17)	0.01 (0.00−0.01)	4.00 (2.00−7.00)	24.14 (15.16−35.70)	0.73 (0.70−0.77)	0.57 (0.26−1.02)
7	0.01 (−2.73−2.77)	0.37 (−3.23−3.99)	0.01 (0.00−0.01)	4.00 (2.00−8.00)	25.40 (15.49−36.73)	0.73 (0.70−0.77)	0.54 (0.25−1.01)
8	−0.03 (−2.83−2.66)	0.63 (−2.93−4.21)	0.01 (0.00−0.01)	5.00 (2.00−8.00)	27.30 (17.00−38.15)	0.73 (0.70−0.77)	0.53 (0.25−0.95)
9	−0.24 (−3.09−2.68)	0.55 (−3.00−4.32)	0.01 (0.00−0.01)	5.00 (3.00−9.00)	29.43 (19.15−40.96)	0.74 (0.70−0.77)	0.51 (0.25−0.91)
10	−0.10 (−2.97−2.86)	0.43 (−3.13−3.96)	0.01 (0.00−0.01)	5.00 (3.00−9.00)	30.38 (19.58−42.22)	0.74 (0.71−0.79)	0.50 (0.23−0.87)
11	−0.20 (−3.16−2.46)	0.19 (−3.74−4.61)	0.01 (0.00−0.01)	6.00 (4.00−10.00)	34.10 (24.02−44.48)	0.75 (0.71−0.79)	0.47 (0.23−0.85)
12	−0.59 (−3.69−1.79)	1.46 (−3.26−5.81)	0.01 (0.00−0.01)	7.00 (5.00−11.00)	35.40 (24.61−45.32)	0.75 (0.71−0.81)	0.48 (0.24−0.85)
	**Eccentric Gaze**	**Efficiency**	**Variance**	**Eccentric Gaze**	**Eccentric Gaze**	**Smooth Pursuit**	
	**Variability Middle**			**Variability Bottom**	**Mean Middle Difference**	**Variance Difference**	
1	2.65 (2.06−3.75)	19.91 (13.03−32.17)	17.09 (10.85−24.84)	0.65 (0.25−1.23)	3.07 (1.36−5.19)	1.59 (0.59−3.80)	
2	2.50 (1.98−3.43)	17.51 (11.42−27.40)	15.55 (9.53−22.75)	0.64 (0.29−1.28)	3.13 (1.45−5.72)	1.34 (0.51−3.34)	
3	2.28 (1.84−2.99)	13.21 (9.44−20.69)	14.02 (8.03−20.70)	0.60 (0.27−1.19)	3.15 (1.48−5.81)	1.26 (0.47−3.25)	
4	2.06 (1.68−2.56)	9.81 (7.58−14.30)	13.62 (7.75−19.82)	0.53 (0.24−1.02)	3.32 (1.52−6.24)	1.22 (0.46−3.18)	
5	2.04 (1.68−2.50)	8.97 (7.24−12.32)	14.30 (8.54−20.62)	0.55 (0.24−1.05)	3.58 (1.67−6.53)	1.30 (0.49−3.32)	
6	2.07 (1.70−2.51)	9.13 (7.34−12.88)	14.20 (8.50−20.55)	0.53 (0.25−1.03)	3.55 (1.61−6.39)	1.34 (0.50−3.67)	
7	2.08 (1.70−2.59)	9.50 (7.51−13.49)	14.58 (8.96−21.02)	0.51 (0.23−1.00)	3.38 (1.59−6.31)	1.36 (0.50−3.63)	
8	2.13 (1.73−2.66)	9.92 (7.77−14.48)	15.02 (9.31−21.41)	0.54 (0.23−1.05)	3.32 (1.54−5.94)	1.55 (0.54−4.27)	
9	2.22 (1.80−2.75)	10.20 (7.96−15.37)	14.61 (8.84−21.06)	0.57 (0.26−1.11)	3.23 (1.52−5.66)	1.80 (0.63−5.49)	
10	2.24 (1.84−2.77)	10.54 (8.16−15.71)	14.41 (8.83−20.86)	0.57 (0.24−1.09)	3.13 (1.51−5.41)	1.85 (0.64−6.15)	
11	2.37 (1.91−2.95)	12.36 (8.70−18.93)	15.26 (9.15−21.36)	0.62 (0.27−1.20)	3.05 (1.41−5.32)	2.28 (0.78−6.74)	
12	2.49 (2.11−3.07)	12.74 (9.45−20.07)	16.10 (10.48−23.50)	0.95 (0.41−1.88)	3.06 (1.50−5.61)	2.47 (0.88−6.87)	

**Table A11:** Horizontal saccade median and interquartile range for the 14 contributing eye movement variables.

**Fixation**	**On**	**Band 2**	**Band 3**	**Band 3**	**Missed**	**Missed Under**
**Number**	**Target**	**Under**	**Over**	**Under**		**Right Side**
15.50 (12.00−18.00)	4.00 (2.50−6.00)	0.50 (0.00−1.50)	2.50 (1.50−4.00)	0.00 (0.00−1.00)	2.00 (1.00−3.50)	0.00 (0.00−1.00)
17.50 (14.00−21.00)	4.50 (2.50−6.50)	1.00 (0.50−2.00)	3.00 (2.00−5.00)	0.50 (0.00−1.50)	2.50 (1.00−4.00)	0.00 (0.00−1.00)
20.50 (16.50−24.00)	4.50 (2.50−6.50)	1.50 (0.50−2.50)	4.00 (2.50−6.00)	0.50 (0.00−2.00)	3.00 (1.50−5.00)	0.50 (0.00−1.00)
23.00 (19.00−27.00)	5.00 (3.00−7.50)	1.50 (0.50−3.00)	4.50 (3.00−7.00)	1.00 (0.00−2.50)	3.00 (1.50−6.00)	0.00 (0.00−1.00)
22.50 (18.00−26.00)	5.00 (2.50−7.50)	1.50 (0.50−3.00)	5.00 (3.00−7.00)	0.50 (0.00−2.00)	2.50 (1.00−5.50)	0.00 (0.00−0.50)
21.00 (16.00−25.00)	4.50 (2.50−7.00)	1.00 (0.50−2.50)	4.50 (2.50−6.50)	0.50 (0.00−1.50)	2.50 (1.00−4.50)	0.00 (0.00−0.50)
19.50 (15.00−24.00)	4.50 (2.50−7.00)	1.00 (0.50−2.50)	4.00 (2.00−6.00)	0.50 (0.00−1.50)	2.00 (1.00−4.00)	0.00 (0.00−0.50)
19.00 (14.50−22.50)	4.50 (2.50−7.00)	1.00 (0.50−2.50)	3.50 (2.00−5.50)	0.50 (0.00−1.50)	1.50 (1.00−3.50)	0.00 (0.00−0.50)
18.00 (14.00−22.00)	4.00 (2.50−6.50)	1.00 (0.50−2.50)	3.50 (2.00−5.50)	0.50 (0.00−1.50)	1.50 (1.00−3.50)	0.00 (0.00−0.50)
17.50 (13.50−21.00)	4.00 (2.50−6.50)	1.00 (0.50−2.50)	3.50 (2.00−5.50)	0.50 (0.00−1.50)	1.50 (1.00−3.50)	0.00 (0.00−0.50)
16.00 (13.00−19.00)	4.00 (2.00−6.00)	1.00 (0.00−2.00)	3.00 (1.50−5.50)	0.50 (0.00−1.00)	2.00 (1.00−3.50)	0.00 (0.00−0.50)
16.00 (12.00−19.50)	3.50 (2.00−5.50)	1.00 (0.25−2.00)	3.00 (1.50−5.00)	0.50 (0.00−1.50)	2.00 (1.00−3.50)	0.00 (0.00−0.50)
**Saccadic **	**Saccadic **	**Saccadic **	**Speed Accuracy**	**Saccadic **	**Saccadic **	**Q Ratio**
**Efficiency**	**Targeting**	**Velocity**	**Trade Off**	**Recovery**	**Amplitude**	
5.30 (4.52−6.39)	9.18 (7.49−11.65)	37.89 (31.68−44.54)	4.05 (3.11−5.04)	1.38 (1.17−1.70)	188.64 (173.94−201.16)	2.42 (2.22−2.71)
5.47 (4.62−6.62)	9.48 (7.69−12.04)	42.51 (34.55−50.75)	4.44 (3.38−5.54)	1.41 (1.19−1.69)	193.31 (179.52−204.09)	2.39 (2.19−2.67)
5.66 (4.76−6.88)	10.02 (8.11−12.58)	49.12 (40.43−57.98)	4.90 (3.72−6.18)	1.45 (1.22−1.79)	197.66 (184.57−207.19)	2.36 (2.16−2.61)
5.65 (4.67−6.91)	10.04 (8.18−12.85)	56.14 (46.59−65.26)	5.63 (4.18−7.07)	1.47 (1.23−1.80)	202.84 (192.27−210.54)	2.26 (2.08−2.50)
5.54 (4.57−6.79)	9.84 (7.89−12.62)	54.55 (45.03−63.57)	5.67 (4.06−7.22)	1.44 (1.20−1.75)	205.62 (196.62−212.27)	2.18 (2.04−2.41)
5.42 (4.52−6.69)	9.96 (7.87−13.17)	50.94 (40.86−60.02)	5.14 (3.59−6.79)	1.38 (1.16−1.68)	204.45 (194.69−211.58)	2.18 (2.03−2.40)
5.38 (4.46−6.56)	10.09 (7.95−13.62)	47.97 (38.45−57.01)	4.89 (3.21−6.52)	1.34 (1.13−1.63)	203.53 (193.06−210.17)	2.18 (2.03−2.41)
5.37 (4.45−6.64)	10.13 (7.91−13.79)	45.76 (36.21−54.43)	4.56 (2.99−6.20)	1.33 (1.12−1.60)	201.51 (190.32−208.71)	2.19 (2.03−2.42)
5.49 (4.54−6.81)	10.19 (8.03−13.84)	44.47 (35.56−52.54)	4.41 (2.89−6.00)	1.37 (1.15−1.68)	200.17 (187.80−207.44)	2.22 (2.05−2.45)
5.51 (4.55−6.80)	10.27 (8.16−14.20)	42.99 (34.22−51.08)	4.22 (2.70−5.66)	1.38 (1.16−1.68)	198.08 (183.46−206.75)	2.24 (2.07−2.48)
5.63 (4.68−6.76)	10.41 (8.20−14.81)	40.08 (32.20−46.94)	3.82 (2.39−5.32)	1.43 (1.18−1.76)	195.00 (178.47−205.50)	2.29 (2.10−2.55)
5.65 (4.81−6.91)	11.05 (8.63−15.37)	39.69 (32.17−47.04)	3.60 (2.39−4.84)	1.51 (1.27−1.77)	191.28 (175.47−202.43)	2.29 (2.13−2.57)

**Table A12:** Vertical saccade median and interquartile range for the 12 contributing eye movement variables.

	**Fixation**	**On**	**Band 2**	**Band 2**	**Band 3**	**Missed**
	**Number**	**Target**	**Over**	**Under**	**Under**	
1	15.00 (12.00−17.50)	3.50 (2.50−5.50)	3.50 (2.00−4.50)	1.00 (0.50−1.50)	0.50 (0.00−1.50)	3.00 (1.62−5.00)
2	17.00 (14.00−20.00)	4.00 (2.50−5.50)	3.50 (2.50−5.00)	1.50 (0.50−2.50)	1.00 (0.00−2.00)	3.50 (2.00−6.00)
3	19.25 (16.00−23.00)	4.50 (3.00−6.00)	4.00 (2.50−5.50)	1.50 (1.00−3.00)	1.00 (0.50−2.50)	4.00 (2.00−6.50)
4	22.00 (19.00−25.00)	5.00 (3.50−7.00)	4.00 (2.50−6.00)	2.00 (1.00−3.50)	1.50 (0.50−3.00)	4.00 (2.00−7.00)
5	22.00 (18.00−25.00)	5.50 (3.50−7.50)	4.00 (2.50−6.00)	2.50 (1.50−4.00)	1.50 (0.50−3.12)	3.00 (1.50−5.00)
6	21.00 (17.00−24.00)	5.50 (3.50−7.50)	4.00 (2.50−5.50)	2.50 (1.00−4.00)	1.50 (0.50−3.00)	2.50 (1.50−4.50)
7	20.00 (16.00−23.00)	5.00 (3.50−7.50)	4.00 (2.00−5.50)	2.50 (1.00−3.50)	1.00 (0.00−3.00)	2.50 (1.00−4.50)
8	19.00 (15.00−22.00)	5.00 (3.00−7.00)	3.50 (2.00−5.50)	2.00 (1.00−3.50)	1.00 (0.00−2.50)	2.00 (1.00−4.50)
9	18.50 (15.00−21.00)	4.50 (3.00−6.50)	3.50 (2.00−5.50)	2.00 (0.50−3.00)	1.00 (0.00−2.50)	2.50 (1.00−4.50)
10	18.00 (14.00−21.00)	4.00 (2.50−6.00)	4.00 (2.50−5.50)	1.50 (0.50−3.00)	1.00 (0.00−2.00)	2.50 (1.00−4.50)
11	17.00 (14.00−20.00)	3.50 (2.00−5.00)	3.50 (2.00−5.00)	1.50 (0.50−2.50)	0.50 (0.00−2.00)	3.00 (1.50−5.00)
12	16.50 (13.00−20.00)	3.00 (1.50−5.00)	3.50 (2.00−5.00)	1.00 (0.50−2.50)	1.00 (0.00−2.00)	3.00 (1.50−5.50)
	**Saccadic**	**Saccadic**	**Saccadic**	**Saccadic**	**Saccadic**	**Q Ratio**
	**Efficiency**	**Targeting**	**Velocity**	**Variance**	**Amplitude**	
1	5.75 (4.85−6.99)	11.08 (8.80−14.06)	38.91 (32.47−46.50)	3.83 (3.18−4.72)	177.18 (161.65−190.94)	2.40 (2.23−2.64)
2	6.01 (4.99−7.56)	11.21 (9.01−14.53)	43.75 (35.75−52.49)	3.90 (3.20−4.98)	181.20 (166.56−194.19)	2.40 (2.22−2.64)
3	6.11 (5.07−7.72)	11.25 (9.17−14.25)	49.04 (40.65−57.89)	3.85 (3.18−4.91)	186.52 (172.66−198.28)	2.35 (2.18−2.57)
4	5.98 (4.97−7.37)	10.77 (8.86−13.26)	54.27 (46.23−62.55)	3.68 (3.08−4.54)	194.40 (182.04−203.60)	2.29 (2.12−2.51)
5	5.72 (4.73−7.04)	9.94 (8.29−12.21)	52.85 (44.62−61.38)	3.46 (2.90−4.23)	197.97 (187.34−205.79)	2.29 (2.10−2.52)
6	5.60 (4.67−6.92)	9.94 (8.25−12.26)	50.01 (41.90−58.68)	3.43 (2.86−4.20)	197.35 (186.49−204.78)	2.31 (2.11−2.55)
7	5.55 (4.61−6.87)	9.90 (8.17−12.25)	48.54 (40.01−57.23)	3.37 (2.82−4.11)	196.71 (185.21−205.11)	2.31 (2.11−2.57)
8	5.59 (4.65−6.88)	10.06 (8.16−12.51)	47.04 (38.73−55.83)	3.36 (2.83−4.17)	196.52 (183.89−205.01)	2.33 (2.12−2.58)
9	5.67 (4.68−7.02)	10.14 (8.22−12.76)	46.56 (37.76−55.15)	3.41 (2.84−4.25)	195.54 (181.95−204.49)	2.33 (2.12−2.60)
10	5.86 (4.78−7.31)	10.31 (8.42−13.34)	45.78 (36.64−55.57)	3.50 (2.88−4.44)	194.62 (180.34−204.37)	2.33 (2.12−2.60)
11	6.13 (5.08−7.69)	11.07 (8.98−14.28)	44.06 (35.19−52.47)	3.73 (3.06−4.79)	189.71 (172.11−201.98)	2.36 (2.13−2.63)
12	6.82 (5.41−8.28)	11.87 (9.52−15.18)	44.94 (34.86−53.89)	4.02 (3.32−5.29)	186.31 (164.71−198.72)	2.37 (2.15−2.64)

**Table A13:** Fixation stability median and interquartile range for the 13 contributing eye movement variables.

**Group**	**Depth**	**Convergence Point**	**Bivariate Contour**	**Gaze Position**	**Gaze Position**	**Gaze Position**	**Gaze Position**
			**Ellipse Analysis**	**1 (Left)**	**1 (Right)**	**3 (Left)**	**3 (Right)**
1	−2.46 (−27.14−23.84)	532.92 (497.97−575.31)	5.88 (5.32−6.51)	63.30 (34.67−79.46)	64.59 (46.33−80.46)	9.48 (2.53−22.07)	8.19 (2.28−20.33)
2	−0.89 (−26.86−26.54)	535.84 (501.58−575.78)	5.48 (4.91−6.20)	66.55 (40.99−84.13)	65.84 (39.93−84.43)	5.13 (0.84−16.50)	5.39 (0.78−16.30)
3	0.35 (−26.60−26.81)	542.62 (506.29−583.75)	5.12 (4.60−5.77)	68.37 (36.39−88.50)	68.85 (38.65−88.40)	2.68 (0.23−12.94)	2.68 (0.27−13.73)
4	−2.43 (−29.59−26.90)	561.46 (524.23−602.94)	4.80 (4.33−5.42)	71.32 (36.68−91.42)	69.87 (33.41−90.87)	1.18 (0.06−8.71)	1.40 (0.11−10.11)
5	−9.16 (−37.34−19.61)	572.25 (534.62−613.11)	4.73 (4.29−5.31)	72.09 (35.07−92.17)	70.35 (33.08−91.53)	1.01 (0.06−8.18)	1.12 (0.06−9.58)
6	−14.47 (−41.46−14.82)	579.24 (539.48−619.12)	4.74 (4.30−5.36)	69.37 (33.73−91.59)	70.79 (35.09−91.74)	1.02 (0.06−9.82)	1.14 (0.06−9.80)
7	−13.89 (−39.90−14.57)	580.67 (542.32−620.18)	4.78 (4.32−5.36)	72.13 (39.19−91.87)	72.32 (37.59−91.97)	1.01 (0.06−7.66)	1.18 (0.11−8.84)
8	−15.31 (−41.21−11.40)	581.11 (543.16−619.54)	4.85 (4.39−5.48)	74.00 (44.48−90.90)	72.71 (40.82−91.45)	1.01 (0.11−7.29)	1.20 (0.11−8.24)
9	−12.85 (−37.57−11.68)	579.51 (540.97−617.26)	4.90 (4.45−5.48)	75.09 (45.13−91.50)	72.29 (41.75−91.04)	1.18 (0.06−7.50)	1.45 (0.11−9.14)
10	−12.10 (−36.38−14.58)	573.18 (538.07−610.91)	4.93 (4.49−5.53)	71.90 (42.21−90.32)	72.34 (43.20−90.24)	1.40 (0.11−8.42)	1.54 (0.11−8.44)
11	−7.53 (−34.24−19.60)	567.55 (531.56−608.90)	5.09 (4.64−5.65)	70.38 (44.96−89.00)	70.53 (42.57−88.24)	1.76 (0.22−8.97)	1.70 (0.22−9.39)
12	−6.56 (−32.08−23.60)	565.35 (528.91−607.56)	5.11 (4.63−5.69)	66.72 (36.36−87.03)	72.43 (44.00−89.12)	2.42 (0.39−9.09)	2.24 (0.34−9.07)
	**Disassociated Tropia**	**Horizontal Targeting**	**Horizontal Targeting**	**Vertical Targeting**	**Vertical Targeting**	**Fixation**	
		**Displacement (Left)**	**Displacement (Right)**	**Displacement (Left)**	**Displacement (Right)**	**Dispersion**	
1	−0.11 (−0.45−0.38)	−0.04 (−0.32−0.29)	−0.01 (−0.28−0.32)	−0.69 (−1.15−−0.19)	−0.54 (−1.04−−0.16)	7.00 (5.41−9.06)	
2	−0.04 (−0.45−0.36)	−0.01 (−0.32−0.32)	0.02 (−0.32−0.34)	−0.62 (−1.04−−0.17)	−0.58 (−1.04−−0.11)	6.68 (4.91−8.90)	
3	−0.06 (−0.48−0.37)	0.01 (−0.32−0.35)	−0.01 (−0.35−0.35)	−0.59 (−1.08−−0.15)	−0.54 (−1.03−−0.06)	6.23 (4.55−8.62)	
4	−0.02 (−0.45−0.42)	−0.03 (−0.37−0.33)	0.02 (−0.34−0.38)	−0.56 (−1.02−−0.10)	−0.54 (−1.04−−0.07)	6.08 (4.37−8.51)	
5	−0.03 (−0.45−0.42)	−0.08 (−0.42−0.27)	0.09 (−0.26−0.45)	−0.55 (−1.05−−0.08)	−0.52 (−1.04−−0.06)	6.02 (4.30−8.37)	
6	−0.04 (−0.48−0.41)	−0.12 (−0.49−0.24)	0.15 (−0.21−0.49)	−0.54 (−1.02−−0.12)	−0.53 (−1.01−−0.09)	5.96 (4.26−8.37)	
7	0.00 (−0.43−0.42)	−0.12 (−0.47−0.23)	0.14 (−0.21−0.48)	−0.50 (−0.96−−0.06)	−0.49 (−0.97−−0.04)	5.89 (4.26−8.28)	
8	0.00 (−0.40−0.41)	−0.15 (−0.47−0.21)	0.15 (−0.19−0.50)	−0.43 (−0.88−0.03)	−0.42 (−0.89−0.03)	5.88 (4.23−8.04)	
9	0.00 (−0.43−0.42)	−0.10 (−0.43−0.23)	0.12 (−0.21−0.47)	−0.39 (−0.84−0.08)	−0.38 (−0.87−0.10)	5.86 (4.28−8.11)	
10	0.01 (−0.42−0.40)	−0.10 (−0.47−0.22)	0.09 (−0.24−0.44)	−0.38 (−0.87−0.10)	−0.38 (−0.85−0.10)	5.84 (4.36−7.96)	
11	−0.01 (−0.44−0.45)	−0.09 (−0.42−0.25)	0.06 (−0.30−0.44)	−0.29 (−0.76−0.21)	−0.30 (−0.77−0.22)	6.03 (4.52−8.07)	
12	−0.05 (−0.45−0.45)	−0.08 (−0.40−0.30)	0.02 (−0.34−0.40)	−0.28 (−0.74−0.30)	−0.22 (−0.74−0.30)	5.78 (4.53−7.93)	

**Table A14:** Calibration median and interquartile range for the 13 contributing eye movement variables.

	Pupil Diameter Mean	Pupil Diameter Difference
1	3.72 (3.34−4.07)	0.11 (0.05−0.21)
2	3.79 (3.43−4.19)	0.11 (0.05−0.20)
3	3.84 (3.45−4.25)	0.12 (0.06−0.21)
4	3.77 (3.36−4.19)	0.12 (0.06−0.21)
5	3.52 (3.15−3.94)	0.12 (0.05−0.21)
6	3.38 (3.02−3.76)	0.11 (0.05−0.20)
7	3.21 (2.87−3.59)	0.11 (0.05−0.20)
8	3.04 (2.74−3.39)	0.11 (0.05−0.19)
9	2.96 (2.64−3.30)	0.11 (0.05−0.20)
10	2.88 (2.57−3.22)	0.10 (0.05−0.19)
11	2.81 (2.48−3.13)	0.12 (0.06−0.21)
12	2.61 (2.38−3.00)	0.09 (0.04−0.18)

## References

[1] Leigh J.R., Zee D.S. (2012). The Neurology of Eye Movements.

[2] Gitchel G., Wetzel P., Baron M. (2012). Pervasive ocular tremor in patients with Parkinson’s disease. Arch. Neurol..

[3] Poole A., Ball L.J., Ghaoui C. (2005). Eye tracking in human-computer interaction and usability research: Current status and future prospects. Encyclopedia of Human-Computer Interaction.

[4] Tenenbaum G., Starkes J.L., Ericsson K.A. (2003). Expert athletes: An integrated approach to decision making. Expert Performance in Sports: Advances in Research on Sport Expertise.

[5] Campbell D., Volkmar F.R. (2013). Normative Data. Encyclopedia of Autism Spectrum Disorders.

[6] Scherf K.S., Sweeney J.A., Luna B. (2006). Brain basis of developmental change in visuospatial working memory. J. Cogn. Neurosci..

[7] Murray N.P., Kubitz K., Roberts C.M., Bolte T., Tyagi A. (2019). An examination of the oculomotor metrics within a suite of digitized eye tracking tests. Vis. Dev. Rehab..

[8] Kullman A., Ashmore R.C., Braverman A., Mazur C., Snapp H., Williams E., Szczupak M., Murphy S., Marshall K., Crawford J. (2021). Normative data for ages 18–45 for ocular motor and vestibular testing using eye tracking. Investig. Otolaryngol..

[9] Mokler A., Fischer B. (1999). The recognition and correction of involuntary prosaccades in an antisaccade task. Exp. Brain Res..

[10] Gould T.D., Bastain T.M., Israel M.E., Hommer D.W., Castellanos F.X. (2001). Altered performance on an ocular fixation task in attention-deficit/hyperactivity disorder. Biol. Psychiatry.

[11] Fukushima J., Hatta T., Fukushima K. (2000). Development of voluntary control of saccadic eye movements I Age-related changes in normal children. Brain Dev..

[12] Kullman A., Ashmore R.C., Braverman A., Mazur C., Snapp H., Williams E., Szczupak M., Murphy S., Marshall K., Crawford J. (2021). Portable eye-tracking as a reliable assessment of oculomotor, cognitive and reaction time function: Normative data for 18–45 year old. PLoS ONE.

[13] Lenzenweger M.F., Driscoll G.A. (2006). Smooth pursuit eye movement and schizotypy in the community. J. Abnorm. Psychol..

[14] Bellmann C., Feely M., Crossland M.D., Kabanarou S.A., Rubin G.S. (2004). Fixation stability using central and pericentral fixation targets in patients with age-related macular degeneration. Ophthalmology.

[15] Pedregosa F., Varoquaux G., Gramfort A., Michel V., Thirion B., Grisel O., Blondel M., Prettenhofer P., Weiss R., Dubourg V. (2011). Scikit-learn: Machine Learning in Python. J. Mach. Learn. Res..

[16] Srivastava T.A. (2015). Basics of Ensemble Learning Explained in Simple English. Analytics Vidhya. http://www.analyticsvidhya.com/blog/2015/08/introduction-ensemble-learning/.

[17] Wood T. (2023). What Is a Random Forest?. https://deepai.org/machine-learning-glossary-and-terms/random-forest.

[18] Yarowsky D. Unsupervised word sense disambiguation rivaling supervised methods. Proceedings of the 33rd Annual Meeting of the Association for Computational Linguistics.

[19] Frost J. (2020). Introduction to Statistics: An Intuitive Guide for Analyzing Data and Unlocking Discoveries.

[20] Kernisan L. 6 Ways That Thinking Changes with Aging (& What to Do About This). Better Health While Aging. https://betterhealthwhileaging.net/how-brain-function-changes-with-normal-cognitive-aging/.

[21] Healthy Aging UCSF Weill Institute for Neurosciences. https://memory.ucsf.edu/symptoms/healthy-aging.

[22] Forstmann B.U., Tittgemeyer M., Wagenmakers E.J., Derrfuss J., Imperati D., Brown S. (2011). The speed-accuracy trade-off in the elderly brain: A structural model-based approach. J. Neurosci..

[23] Karatekin C. (2007). Eye tracking studies of normative and atypical development. Dev. Rev..

[24] Sobolev M., Gullapalli B.T., Rahman T. (2021). Advancing the Science of Digital Biomarkers. Proceedings of the 2021 Workshop on Future of Digital Biomarkers.

[25] Wang T., Azad T., Rajan R. (2021). The Emerging Influence of Digital Biomarkers on Healthcare. https://rockhealth.com/reports/the-emerging-influence-of-digital-biomarkers-on-healthcare/.

[26] Coravos A., Khozin S., Mandl K.D. (2019). Developing and adopting safe and effective digital biomarkers to improve patient outcomes. npj Digit. Med..

[27] Paolotti D., Shah U., Edelstein M., Neto O.L., Kostkova P., Wood C. Digital health innovation: From proof of concept to public value. Proceedings of the 9th International Conference on Digital Public Health (DPH’ 2019).

[28] Chernyak I., Chernyak G., Bland J.K.S., Rahier P.D.P. (2021). Important Considerations of Data Collection and Curation for Reliable Benchmarking of End-User Eye-Tracking Systems. Proceedings of the ACM Symposium on Eye Tracking Research and Applications (ETRA ‘21 Full Papers).

[29] Goodwin T.R., Harabagiu S.M. (2016). Medical Question Answering for Clinical Decision Support. Proceedings of the 25th ACM International on Conference on Information and Knowledge Management (CIKM ‘16).

